# Normative modelling of brain morphometry across the lifespan with CentileBrain: algorithm benchmarking and model optimisation

**DOI:** 10.1016/S2589-7500(23)00250-9

**Published:** 2024-03

**Authors:** Ruiyang Ge, Yuetong Yu, Yi Xuan Qi, Yu-nan Fan, Shiyu Chen, Chuntong Gao, Shalaila S Haas, Faye New, Dorret I Boomsma, Henry Brodaty, Rachel M Brouwer, Randy Buckner, Xavier Caseras, Fabrice Crivello, Eveline A Crone, Susanne Erk, Simon E Fisher, Barbara Franke, David C Glahn, Udo Dannlowski, Dominik Grotegerd, Oliver Gruber, Hilleke E Hulshoff Pol, Gunter Schumann, Christian K Tamnes, Henrik Walter, Lara M Wierenga, Neda Jahanshad, Paul M Thompson, Sophia Frangou, ENIGMA Lifespan Working Group

**Affiliations:** Djavad Mowafaghian Centre for Brain Health, University of British Columbia, Vancouver, BC, Canada (R Ge PhD, Y Yu BSc, Y X Qi BSc, Y-n Fan BSc, S Chen BSc, C Gao BSc, Prof S Frangou MD); Department of Psychiatry, Icahn School of Medicine at Mount Sinai, New York, NY, USA (Prof S Frangou, S S Haas PhD, F New MA); Netherlands Twin Register, Department of Biological Psychology, Vrije Universiteit, Amsterdam, Netherlands (Prof D I Boomsma PhD); Centre for Healthy Brain Ageing, University of New South Wales, Sydney, NSW, Australia (Prof H Brodaty DSc); Department of Complex Trait Genetics, Center for Neurogenomics and Cognitive Research, Vrije Universiteit, Amsterdam, Netherlands (R M Brouwer PhD); Center for Brain Science, Harvard University, Cambridge, MA, USA (Prof R Buckner PhD); Centre for Neuropsychiatric Genetics and Genomics, Cardiff University, Cardiff, Wales, UK (X Caseras PhD); Groupe d’Imagerie Neurofonctionnelle—Institut des Maladies Neurodégénératives, Université de Bordeaux, CNRS UMR 5293, Bordeaux, France (F Crivello PhD); Erasmus School of Social and Behavioural Sciences, Erasmus University Rotterdam, Rotterdam, Netherlands (Prof E A Crone PhD); Division of Mind and Brain Research, Department of Psychiatry and Psychotherapy, Charité-Universitätsmedizin Berlin, Berlin, Germany (Prof S Erk MD, Prof H Walter MD); Language and Genetics Department, Max Planck Institute for Psycholinguistics, Nijmegen, Netherlands (Prof S E Fisher Dphil); Departments of Human Genetics, Psychiatry and Cognitive Neuroscience, Donders Institute for Brain, Cognition and Behaviour, Radboud University Medical Center, Nijmegen, Netherlands (Prof B Franke PhD); Department of Psychiatry and Behavioral Sciences, Boston Children’s Hospital, Boston, MA, USA (Prof D C Glahn PhD); Department of Psychiatry and Psychotherapy, University of Münster, Münster, Germany (Prof U Dannlowski MD, D Grotegerd PhD); Section for Experimental Psychopathology and Neuroimaging, Department of General Psychiatry, Heidelberg University, Heidelberg, Germany (Prof O Gruber MD); Department of Experimental Psychology, Helmholtz Institute, Utrecht University, Utrecht, Netherlands (Prof H E Hulshoff Pol PhD); Centre for Population Neuroscience and Stratified Medicine, Institute for Science and Technology of Brain-inspired Intelligence, Fudan University, Shanghai, China (Prof G Schumann MD); PONS Centre, Department of Psychiatry and Clinical Neuroscience, CCM, Charite Universitätsmedizin Berlin, Berlin, Germany (Prof G Schumann); Department of Psychology, University of Oslo, Oslo, Norway (Prof C K Tamnes PhD); Brain and Development Research Center, Leiden University, Leiden, Netherlands (L M Wierenga PhD); Mark and Mary Stevens Neuroimaging and Informatics Institute, Keck School of Medicine of USC, University of Southern California, Marina del Rey, CA, USA (N Jahanshad PhD, Prof P M Thompson PhD)

## Abstract

The value of normative models in research and clinical practice relies on their robustness and a systematic comparison of different modelling algorithms and parameters; however, this has not been done to date. We aimed to identify the optimal approach for normative modelling of brain morphometric data through systematic empirical benchmarking, by quantifying the accuracy of different algorithms and identifying parameters that optimised model performance. We developed this framework with regional morphometric data from 37 407 healthy individuals (53% female and 47% male; aged 3–90 years) from 87 datasets from Europe, Australia, the USA, South Africa, and east Asia following a comparative evaluation of eight algorithms and multiple covariate combinations pertaining to image acquisition and quality, parcellation software versions, global neuroimaging measures, and longitudinal stability. The multivariate fractional polynomial regression (MFPR) emerged as the preferred algorithm, optimised with non-linear polynomials for age and linear effects of global measures as covariates. The MFPR models showed excellent accuracy across the lifespan and within distinct age-bins and longitudinal stability over a 2-year period. The performance of all MFPR models plateaued at sample sizes exceeding 3000 study participants. This model can inform about the biological and behavioural implications of deviations from typical age-related neuroanatomical changes and support future study designs. The model and scripts described here are freely available through CentileBrain.

## Introduction

Normative modelling is a class of statistical methods to quantify the degree to which an individual-level measure deviates from the pattern observed in a normative reference population. Normative modelling of neuroimaging phenotypes has mostly focused on brain morphometry given the wide availability of structural MRI data,^1–4^ with extensions into diffusion MRI in the past couple of years.^5^ Normative modelling is emerging as a promising new approach to the investigation of brain alternations in neuropsychiatric disorders.^6–11^ However, the value of normative models as research, and potentially clinical, tools relies on their methodological robustness, which has yet to be empirically investigated.

Available normative modelling studies employ a range of linear, non-linear, and Bayesian algorithms that reflect researchers’ preferences.^1–13^ At present, there is no systematic comparative evaluation of the performance of these algorithms and no empirical determination of the key parameters that could influence model performance. For example, the minimum sample size necessary for reliable normative estimates of brain morphometric measures has not been established and, with few exceptions,^1–3,13^ the size of the samples used for the normative reference population is small to modest (range 145–870 people).^6–10,14,15^

To address this important knowledge gap, the aim of this study was to identify the optimal approach for the normative modelling of brain morphometric data through systematic empirical benchmarking. Specifically, the aim was to quantify the accuracy of the different algorithms and identify those parameters that optimise model performance.

## Methods

### Samples

We collated de-identified data from 87 datasets from Europe, Australia, the USA, South Africa, and east Asia (appendix 1 p 2; appendix 2). Data use aligned with the policies of the ENIGMA Lifespan Working Group, and the policies of individual studies and national repositories. On the basis of the information provided in each dataset, data were further selected to include high-quality neuroimaging measures (appendix 1 p 3) from participants who did not have psychiatric, medical, and neurological morbidity and cognitive impairment at the time of scanning. Only scans acquired at baseline were included from datasets with multiple scanning assessments. The study design conformed with STROBE guidelines. Normative models are distinguished into reference models, derived from a sample considered representative of a population in a geographical region at a specific period, and standard models, derived from healthy individuals aiming to represent a healthy pattern of age-related changes. Given the nature of our samples, the models developed are standard models.

### Brain morphometry

Acquisition protocols and scanner vendors varied across datasets (appendix 2). Morphometric feature extraction from whole-brain T_1_-weighted images was implemented with the standard pipelines in the FreeSurfer image analysis suite (appendix 2) to yield global measures of total intracranial volume, mean cortical thickness, and total surface area, as well as measures of cortical thickness and cortical surface area from the 68 Desikan-Killiany atlas regions and 14 subcortical volumetric measures based on the Aseg atlas. Sex-specific normative models were developed separately for each of the 150 regional morphometry measures to accommodate sex differences in brain morphometry.^16^ Sex (ie, male or female) was determined by self-report. We explored the clustering of the brain morphometry data by geographical regions and did not identify region-specific clusters (appendix 1 p 2).

### Optimisation of normative models

**Figure 1:**
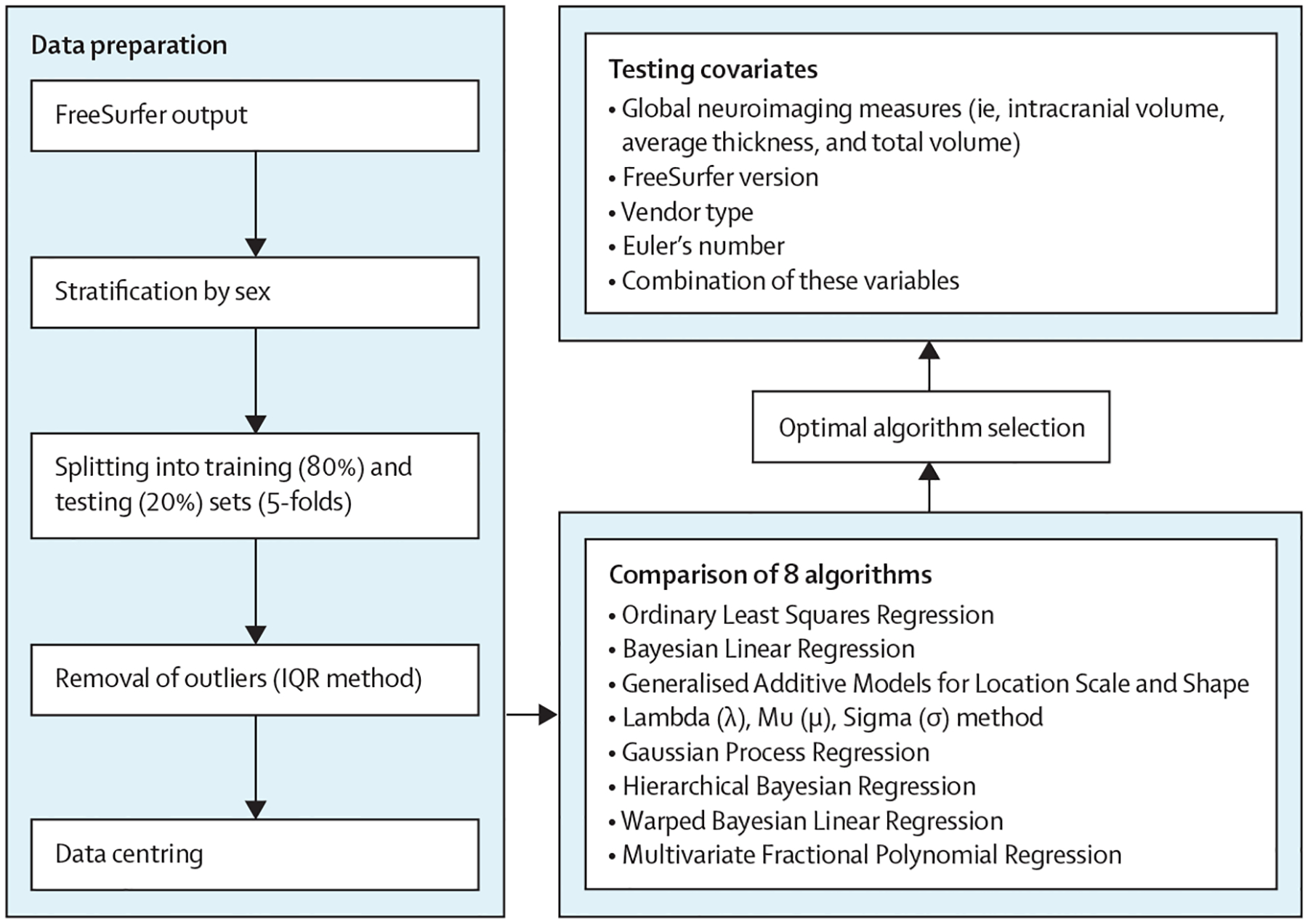
Flowchart of normative model optimisation The study sample was stratified by sex and then split into training (80%) and testing (20%) datasets, followed by outlier removal, and mean-centring. Normative models were generated through eight different algorithms and compared in terms of accuracy and computational efficiency. Explanatory variables were added to identify the appropriate combination for optimal model performance.

The procedures used to generate optimised sex-specific models for each brain morphometric measure are illustrated in figure 1 and consisted of data preparation, algorithm selection, and model optimisation.

#### Data preparation

Sex-specific subsamples of the study sample were split into a training subset (80%) and a test subset (20%), through stratified randomisation by scanning site and age. Data within the training and testing subset were mean-centred after extreme values, defined as any values greater than 1·5 times the IQR,^17^ in each subset were identified and removed.

#### Algorithm selection

The data for each morphometric measure were analysed with the following algorithms: (1) ordinary least squares regression (OLSR), implemented with the lm function in R: this is a linear regression model that aims to minimise the sum of squared differences between the observed and predicted values; (2) Bayesian linear regression (BLR), implemented with the stan package in R: this is a linear model in which the outcome variable and the model parameters are assumed to be drawn from a probability distribution; (3) generalised additive models for location, scale, and shape (GAMLSS), implemented with the caret package in R: this framework can model heteroskedasticity, non-linear effects of variables, and hierarchical structure of the data; (4) parametric Lambda (λ), Mu (μ), Sigma (σ) method (LMS), implemented with the gamlss package in R:^15^ this subclass of GAMLSS assumes that the outcome variable follows the Box-Cox Cole and Green distribution; (5) Gaussian process regression (GPR), implemented with the kernlab package in R and the sigest function for estimating the hyperparameter sigma: this is a non-parametric regression model that follows Bayesian principles; (6) warped Bayesian linear regression (WBLR),^18^ implemented with the “PCNtoolkit” in Python following authors’ recommendations: this framework is based on Bayesian linear regression with likelihood warping; (7) hierarchical Bayesian regression (HBR),^10,12^ implemented with the PCNtoolkit in Python: this approach also uses Bayesian principles and is considered particularly useful when variance from multiple hierarchical levels is present, including the scanning protocol or site effects; (8) multivariate fractional polynomial regression (MFPR), implemented with the mfp package in R and the closed test procedure (known as RA2) to select the most appropriate fractional polynomial: this algorithm enables the determination of the functional form of a predictor variable by testing a broad family of shapes and multiple turning points while providing a good fit at the extremes of the covariates.

The potential effect of site on performance was addressed both by handling site as a random factor and by site-harmonisation using ComBat-GAM^19^ and then comparing the resulting models.

All models were sex-specific. Each sex-specific subsample was divided into the training set (80%) and the testing set (20%) while maintaining the same proportional representation of the sites in the total sample. There was no overlap of participants contributing to the training and the testing sets of each sex-specific subsample. The models were trained with five-fold cross-validation (5F-CV) in the corresponding sex-specific training subset, with age being the only explanatory variable. Model parameters were tested in the corresponding sex-specific test subset. In each cross-validation, 80% of the sample was used to train the model and 20% was used to test the model parameters. The mean absolute error (MAE), which is the average of the absolute differences (ie, errors) between the predicted and the observed data, was averaged across cross-validations and served as the main measure of model performance, supplemented by the root mean square error (RMSE), which is the standard deviation of the prediction errors, and was also averaged across cross-validations and by explained variance. The computational efficiency of each model was assessed through the central processing unit (CPU) time of the supercomputing infrastructure of the Icahn School of Medicine at Mount Sinai.

#### Model optimisation

Model optimisation involved the evaluation of improvements in the MAE (and RMSE and explained variance) by adding the following explanatory variables: global neuroimaging measures (ie, intracranial volume, mean cortical thickness, or mean cortical surface area, as appropriate), and both linear and non-linear contributions from these variables were considered; scanner vendor type; FreeSurfer version; Euler’s number for scan quality; and combinations of these variables. Each model was trained through 5F-CV in the corresponding sex-specific training subset and then tested in the corresponding sex-specific test subset. Variables that significantly improved performance were retained.

Across regional morphometric measures (and separately in males and females), the MAEs and RMSEs of the optimised models generated by each algorithm were concatenated as a single vector to enable pairwise comparisons between algorithms. False discovery rate (FDR) correction for multiple testing was used and results were considered significant at p_fdr_<0·05 across comparisons. Upon completion of data preparation, algorithm selection, and model optimisation, optimised sex-specific and region-specific models were defined on the basis of the best-performing algorithm and covariate combination. The normative deviation score for each region^4,11^ was defined as:

$$Z=\frac{Y-\hat{Y}}{RMSE_{m}}$$

where ŷ is the predicted value, y is the observed value, and RMSE_m_ is the value in the pretrained model.

### Sensitivity analyses

The study sample was partitioned into 75 sex-specific random subsets consisting of 200–15 000 participants in increments of 200. The robustness of the optimised sex-specific and region-specific models to sample size in terms of MAE and RMSE was assessed in each partition using 5F-CV.

Model accuracy could be influenced by the sample’s age range and by distinct challenges encountered in scanning different age groups, such as higher levels of motion in paediatric than adult populations.^20^ Accordingly, the study sample was divided into nine sex-specific age-bins (ie, aged ≤10 years; aged <10 years to ≤20 years; aged <20 years to ≤30 years; aged <30 years to ≤40 years; aged <40 years to ≤50 years; aged <50 years to ≤60 years; aged <60 years to ≤70 years; aged <70 years to ≤80 years; aged <80 years to ≤90 years). The MAE and RMSE of each optimised sex-specific and region-specific model were estimated in each age bin with 5F-CV. Subsequently, Pearson’s correlation coefficients were computed between the MAE and RMSE values of the models within each sex-specific age bin with those derived from the sex-specific subset of the entire sample. Before computing Pearson’s correlation coefficients, we verified the assumption of linearity through the Kolmogorov–Smirnov tests and illustrated this in scatter plots between the MAE and RMSE values of the models within each sex-specific age bin and those derived from the sex-specific subset of the entire sample (appendix 1 p 15).

As the GAMLSS algorithm is particularly popular for normative modelling,^21^ we did additional sensitivity analyses for different GAMLSS models and software packages (appendix 1 pp 16–20).

The Southwest Longitudinal Imaging Multimodal Study (SLIM) and the Queensland Twin Adolescent Brain Study (QTAB) were used to test the longitudinal stability of the optimal normative models. There is no participant overlap between the SLIM and QTAB studies and between either dataset and the sample used for model development. The SLIM dataset includes 118 healthy individuals (59 females and 59 males; age range 17–22 years for the baseline scans and 19–25 years at follow-up scans) who were rescanned with a mean interval of 2·35 years. The QTAB dataset includes 259 healthy individuals (129 females and 130 males; sample age range 9–14 years for the baseline scans and 10–16 years at follow-up scans) who were rescanned with a mean interval of 1·76 years. In these datasets, sex (ie, male or female) was also determined by self-report.

### Relevance of normative models of brain morphometry for mental illness

We tested whether normative brain regional Z-scores have an advantage over the observed morphometric measures in predicting diagnostic status and symptom severity using psychosis as an example. For this test, we downloaded and parcellated (with FreeSurfer version 7.1.0) T_1_-weighted images from the repository of the Human Connectome Project-Early Psychosis Study (HCP-EP). The HCP-EP cohort comprises 91 individuals with early psychosis and 57 healthy individuals (total sample 48 females and 100 males; age range 16·67–35·67 years). Sex (ie, male or female) was determined by self-report.^22^ Each of the algorithms examined here were then applied to generate brain regional Z-scores in the HCP-EP cohort.

For diagnostic status prediction, and for each algorithm, the regional Z-scores and the observed neuromorphometric data were entered into separate support vector classification (SVC) models with a linear kernel from the scikit-learn package (version 1.2.2) following established procedures.^23^ The area under the receiver operating characteristic curve (AUC), averaged across all folds within a 5F-CV framework repeated 100 times, was used to evaluate the classification accuracy of each SVC model. Statistical significance was established by comparing the averaged AUC of each model to a null distribution generated from a model trained on 1000 random permutations of the diagnostic labels (ie, a patient or healthy individual in the HCP-EP cohort). To compare the classification accuracy of the SVC models using the regional Z-scores with the SVC model using the observed neuromorphometric data, we calculated pairwise Δ_*AUC*_ and we tested whether they exceeded chance probability compared with a null distribution using permutation.

For the prediction of symptom severity, and for each algorithm, regional Z-scores and the observed neuromorphometric data were entered into separate ridge regression models with 100 repeats of 5F-CV to predict the psychosis score of the Positive and Negative Syndrome Scale^24^ in the HCP-EP study patients. The MAE of each model, averaged across folds, was used as the performance metric. Within each fold, we applied principal component analysis to reduce the dimensionality of the brain regional measures to the first ten principal components that explained at least 90% of the variance. To compare the predictive accuracy of the regression models using the Z-scores to the model using the observed neuromorphometric data, we calculated pairwise Δ_*MAE*_ and followed the same procedures as for the classification models. In the case of predictive accuracy, permutations involved shuffling the Positive and Negative Syndrome Scale scores of the HCP-EP cohort.

### Role of the funding source

The funders of the study had no role in the study design, data collection, analysis, and interpretation, in the writing of the manuscript, and the decision to submit. All authors had full access to all the data in the study and agreed to submit for publication.

## Results

A total of 37 407 healthy individuals from 87 datasets from 20 countries were included in this study. This sample consisted of 19 964 females and 17 443 males.

**Figure 2:**
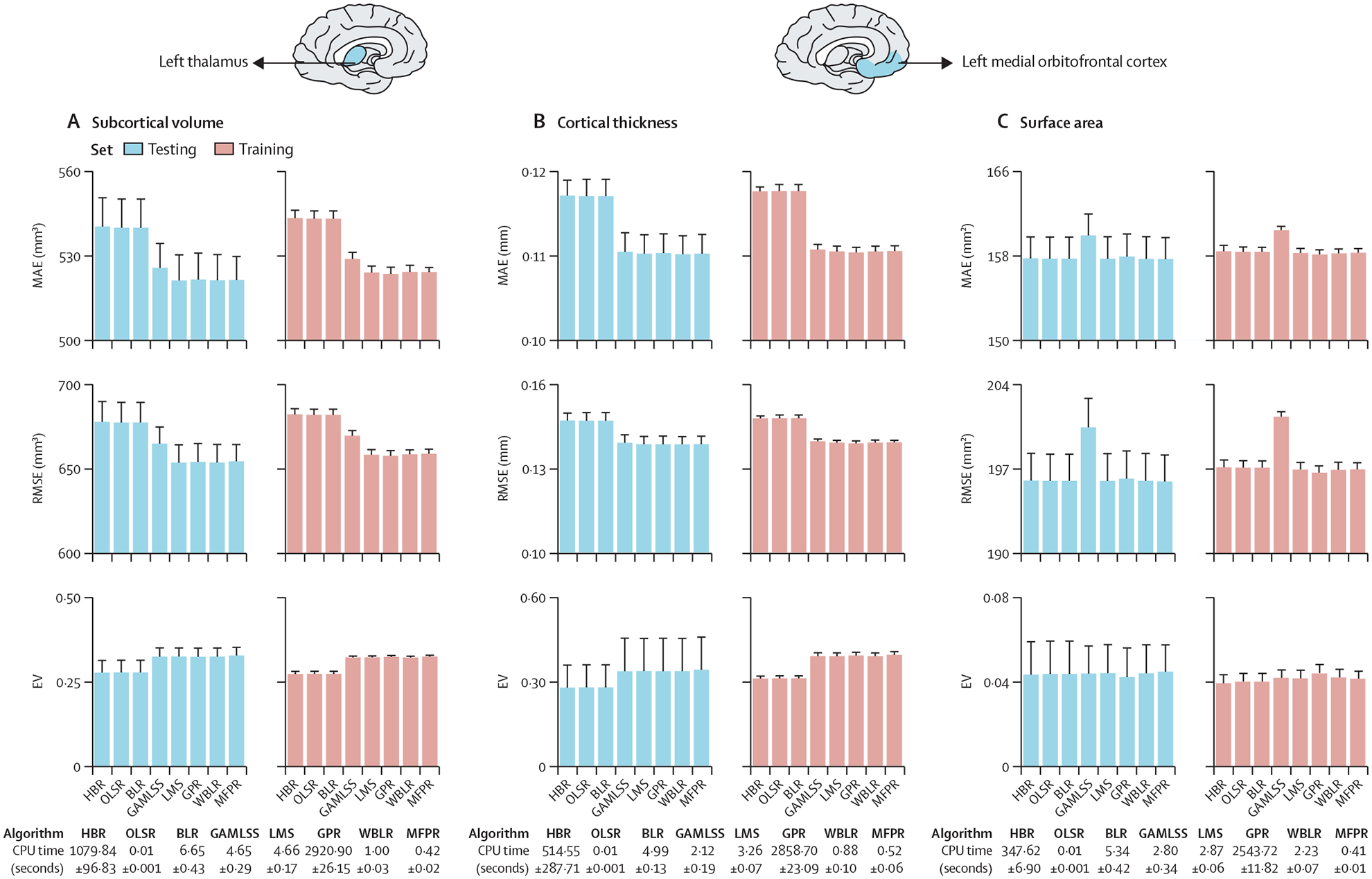
Illustrative examples of comparative algorithm performance Algorithm performance for each regional morphometric measure was assessed separately in males and females with the MAE, RMSE, EV, and CPU time. The MAE, RMSE, EV, and CPU times of the models for left thalamic volume (A), the left medial orbitofrontal cortical thickness (B), and left medial orbitofrontal cortical surface area (C) as exemplars here for females and in appendix 1 (p 4) for males. The pattern identified was the same across all region-specific models and in both sexes (appendix 1 pp 5–6). Note that scales on y axes differ between plots. BLR=Bayesian linear regression. CPU=central processing unit. EV=explained variance. GAMLSS=generalised additive models for location, scale, and shape. GPR=Gaussian process regression. HBR=hierarchical Bayesian regression. LMS=Lambda (λ), Mu (μ), Sigma (σ) method. MAE=mean absolute error. MFPR=multivariate fractional polynomial regression. OLSR=ordinary least squares regression. RMSE=root mean square error. WBLR=warped Bayesian linear regression.

The MAE, RMSE, explained variance, and CPU time of the models for the left thalamic volume and left medial orbitofrontal cortical thickness and surface area in females are shown as exemplars (figure 2; the corresponding data for males is in appendix 1 p 4). The pattern was the same for all regions across sex-specific models (appendix 1 pp 5–6; appendix 3). Across all models, the OLSR and MFPR had the shortest CPU times (less than 1 s) whereas GPR had the longest (25–60 min). Across all sex-specific and region-specific models, the LMS, GPR, WBLR, and MFPR had comparable values for MAE, RMSE, and explained variance that were statistically better at P_FDR_<0·05 than those for GAMLSS, BLR, OLSR, and HBR. Accordingly, the MFPR emerged as the preferred algorithm given its combined advantages in accuracy and computational efficiency.

**Figure 3:**
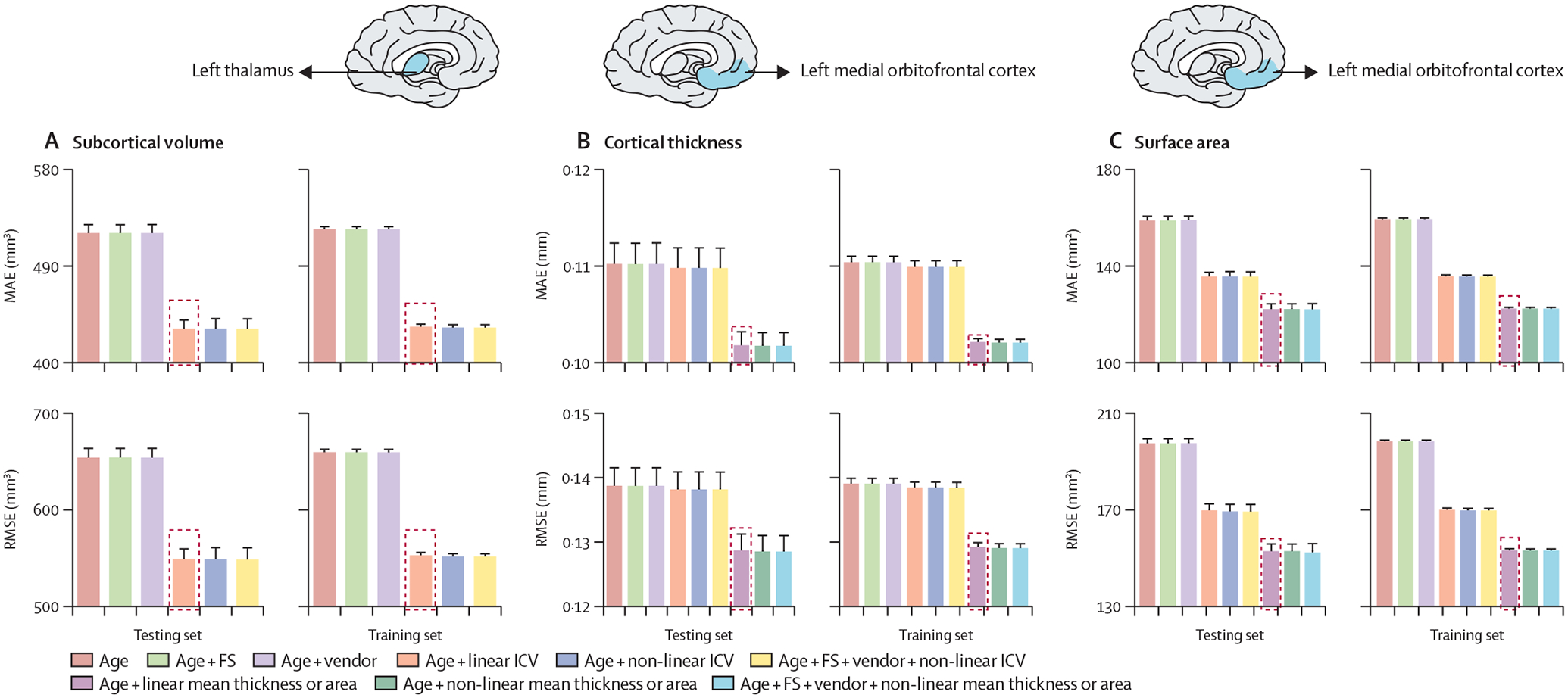
Illustrative examples of the performance of MFPR-derived models as a function of explanatory variables For each regional morphometric measure, sex-specific models derived from all algorithms were trained and tested using nine different covariate combinations that included effects of age, FS version, Euler’s number, scanner vendor, ICV, and global estimates of mean cortical thickness or total area. The MAE and RMSE of models for left thalamic volume (A), the left medial orbitofrontal cortical thickness (B), and left medial orbitofrontal cortical surface area (C) derived from MFPR for females are presented as exemplars; the optimal variable combination is marked with a dashed frame. The corresponding data for males are presented in appendix 1 (p 7). The data for other regions are shown in appendix 1 (pp 8–12). In both sexes, the pattern identified was identical for all region-specific models. Note that scales on y axes differ between plots. FS=FreeSurfer. ICV=intracranial volume. MAE=mean absolute error. MFPR=multivariate fractional polynomial regression. RMSE=root mean square error.

We considered the following covariates in all models: scanner vendor, Euler’s number, FreeSurfer version, and global neuroimaging measures (ie, intracranial volume, mean cortical thickness, or mean cortical surface area, as indicated) and their linear and non-linear combinations. We illustrate the effects of the covariates for the MFPR-derived models of the left thalamic volume and left medial orbitofrontal cortical thickness and surface area in females (figure 3; the corresponding data for males are in appendix 1 p 7). The same pattern was observed for all regions across sex-specific MFPR models (appendix 1 pp 8–9; appendix 4). The effect of the scanner, Euler’s number (appendix 1 p 3), and FreeSurfer version on model performance was small, whereas the opposite was the case for the global neuroimaging measures. Therefore, optimised models included age and global neuroimaging measures (ie, intracranial volume, mean cortical thickness, or total cortical surface area, as indicated).

**Figure 4:**
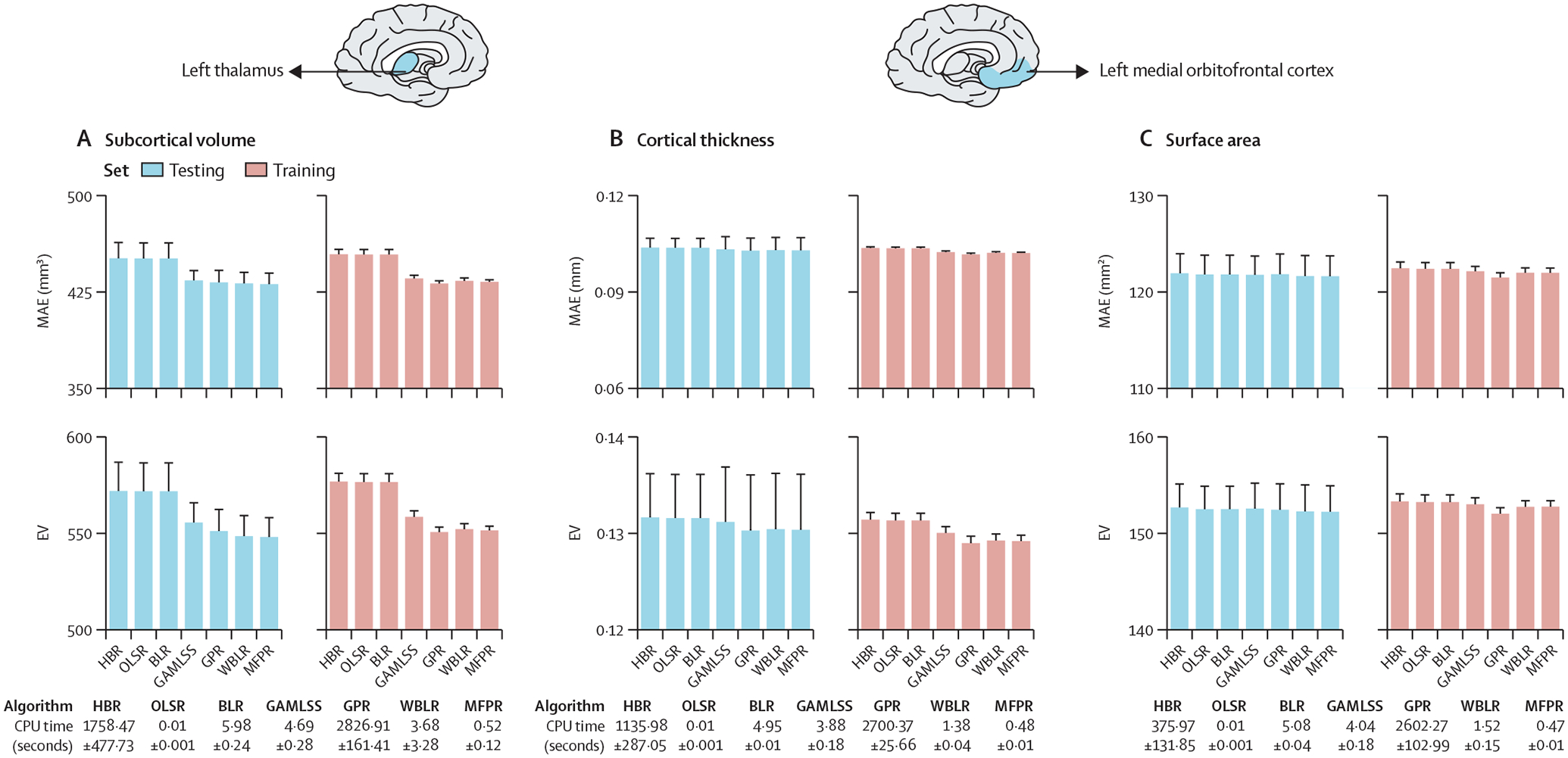
Illustrative examples of the comparative performance of optimised models derived from OLSR, BLR, HBR, GPR, GAMLSS, WBLR, and MFPR Region-specific models with the optimised covariate combination were estimated in males and females separately with OLSR, BLR, HBR, GPR, GAMLSS, WBLR, and MFPR. Model performance was assessed in terms of MAE, RMSE, and CPU time. The MAE, RSME, and CPU time of the models for left thalamic volume (A), the left medial orbitofrontal cortical thickness (B), and left medial orbitofrontal cortical surface area (C) in females are presented as exemplars and in appendix 1 (p 10, figure S9) for males. Note that scales on y axes differ between plots. BLR=Bayesian linear regression. CPU=central processing unit. GAMLSS=generalised additive models for location, scale, and shape. GPR=Gaussian process regression. HBR=hierarchical Bayesian regression. MAE=mean absolute error. MFPR=multivariate fractional polynomial regression. OLSR=ordinary least squares regression. RMSE=root mean square error. WBLR=warped Bayesian linear regression.

We then compared the MAE, RMSE, and CPU time for each of the sex-specific and region-specific optimised models derived from the other algorithms. Statistical comparison of the models from each algorithm at P_FDR_<0·05 indicated comparable performance for the optimised MFPR-derived, WBLR-derived, and GPR-derived models that outperformed the optimised models derived from the other algorithms. We illustrate these findings for females in figure 4 using the left thalamic volume and left medial orbitofrontal cortical thickness and surface area as exemplars (the corresponding data in males and for all other regions are in appendix 1 pp 10–12 and appendix 5). In addition to retaining their accuracy, the MFPR-derived models remained the most computationally efficient, with CPU times of less than a second. Accordingly, we define the optimal models as the sex-specific and region-specific models that were based on the MFPR algorithm with non-linear fractional polynomials of age and linear effects of the appropriate global neuroimaging measure (ie, intracranial volume for models of regional subcortical volumes, mean cortical thickness for models of regional cortical thickness, and total cortical surface area for models of regional cortical surface area).

**Figure 5:**
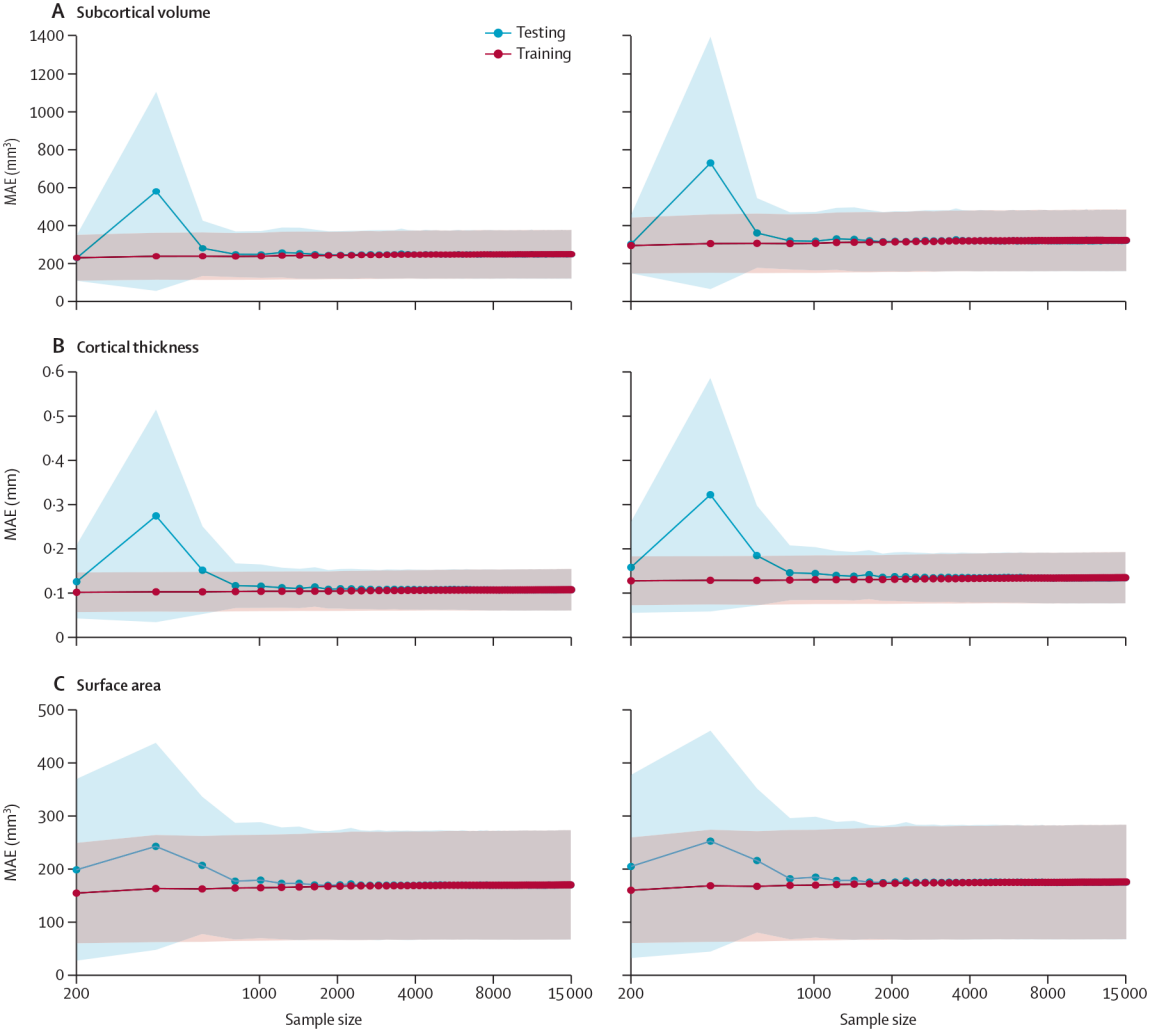
Performance of region-specific MFPR-derived models as a function of sample size Models for each regional morphometric measure were estimated in random sex-specific subsets of 200–15 000 participants, in increments of 200, generated from the study sample. Each line represents the values of the MAE or RMSE derived from the optimised MFPR models of all regional morphometric measure as a function of sample size; shadowed area represents the SD. The pattern identified was identical in both sexes. The data for females are shown here and for males in appendix 1 (p 13). Note that scales on y axes differ between plots. MAE=mean absolute error. MFPR=multivariate fractional polynomial regression. RMSE=root mean square error.

The MAE and RMSE values of the optimised MFPR-derived sex-specific and region-specific models plateaued at a sample size of approximately 3000 participants (figure 5 for females; appendix 1 p 13 for males).

**Figure 6:**
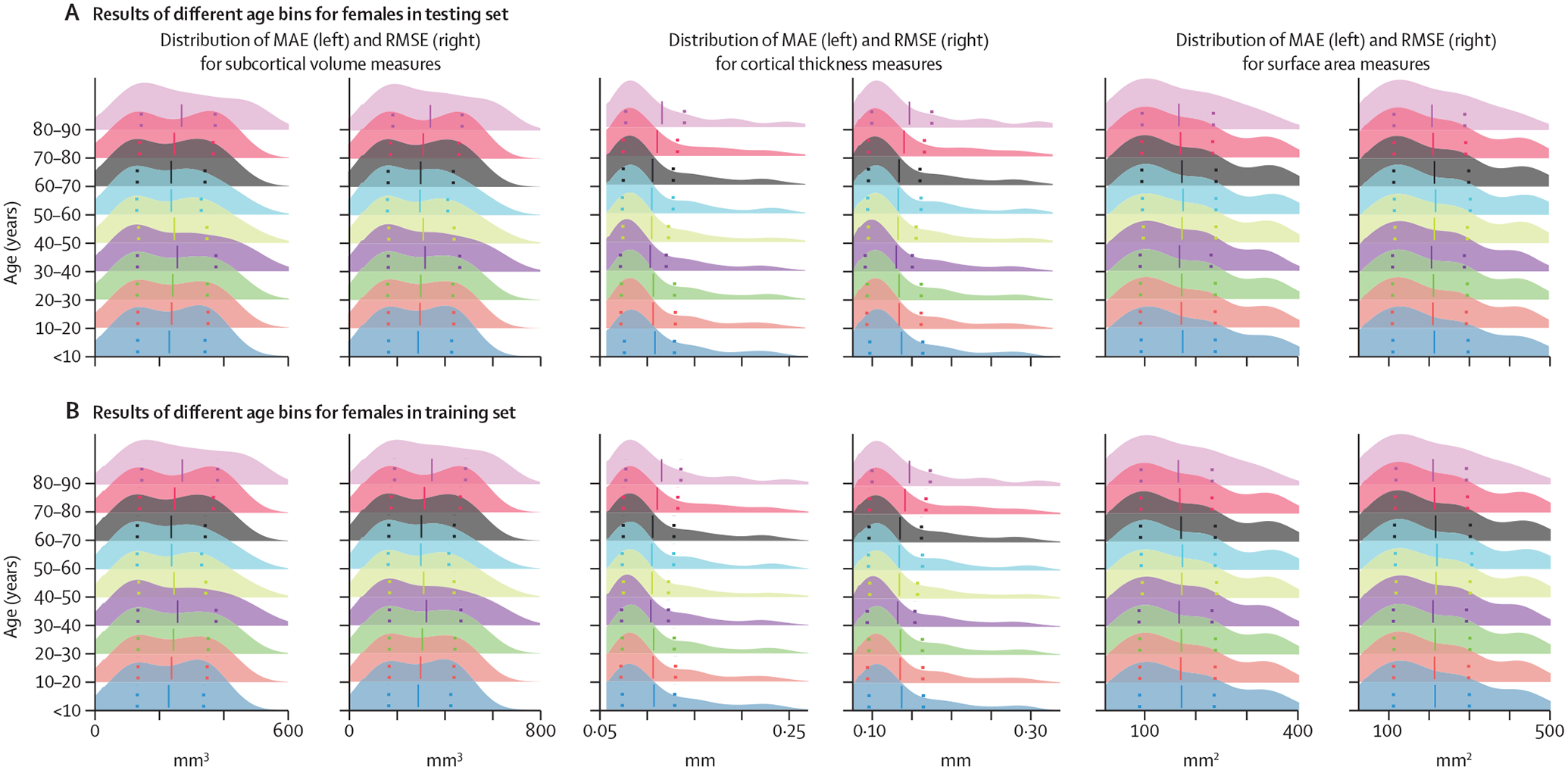
Performance of region-specific models in distinct age bins Sex-specific and region-specific models of all morphometric measures for different age bins were estimated by partitioning the sex-specific training and testing subsets of the study sample into nine age bins (ie, aged ≤10 years; aged <10 years to ≤20 years; aged <20 years to ≤30 years; aged <30 years to ≤40 years; aged <40 years to ≤50 years; aged <50 years to ≤60 years; aged <60 years to ≤70 years; aged <70 years to ≤80 years; aged <80 years to ≤90 years). Details are provided in appendix 5. The figure presents the distribution of the MAE and the RMSE across all region-specific models in females in the training (A) and testing (B) subset. The pattern was identical in both sexes and the results for males are presented in appendix 1 (p 14). Note that scales on y axes differ between plots. MAE=mean absolute error. RMSE=root mean square error.

The MAE and RMSE values of the optimised MFPR-derived sex-specific and region-specific models in each of the nine age bins are presented in figure 6 for females (appendix 1 p 14 and appendix 6 for males). Across all age bins, the correlation coefficient between the MAE or RMSE values of the sex-specific and region-specific models obtained from the full study sample and MAE or RMSE values of the corresponding models estimated in each age bin were all greater than 0·98, suggesting the robustness of the model accuracy across all age groups.

Comparison of different GAMLSS models and software supported the superiority of the choice reported here compared with other alternatives (appendix 1 pp 16–20).

The performance of the OLSR, BLR, GAMLSS, WBLR, HBR, and MFPR models were compared when the site was modelled either as a random factor or by harmonisation with ComBat-GAM. These comparisons excluded the LMS as it does not accommodate multiple explanatory variables and GPR because it assumes only continuous variables. The top-performing algorithm when the site was used as a random effect was still the MFPR, followed closely by WBLR. Furthermore, the model performance of the MFPR algorithm in terms of MAE was similar regardless of how site was handled (details in appendix 1 p 21).

**Figure 7:**
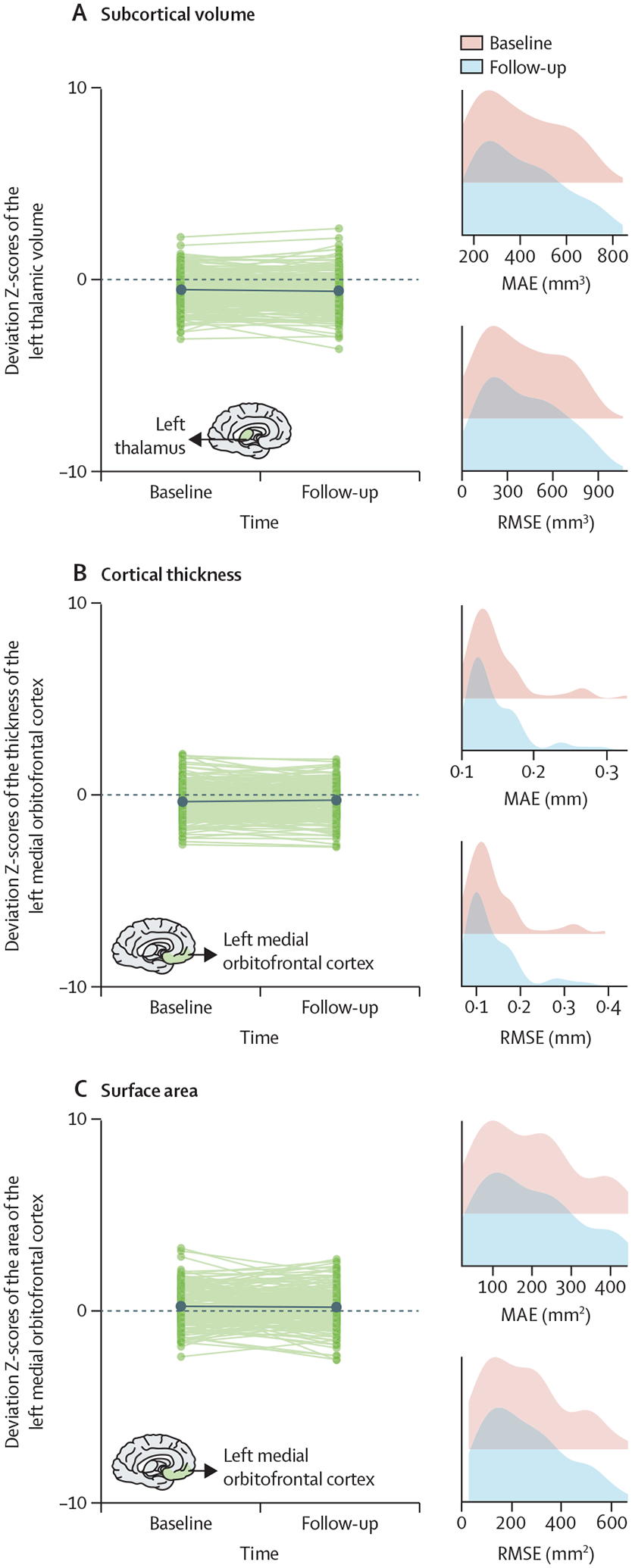
Stability of the normative deviation scores (Z-scores) in longitudinal neuroimaging data We illustrate the stability of the optimised MFPR-derived models over an average interval of 2 years in data from the SLIM and QTAB study samples using the left thalamic volume (A), the left medial orbitofrontal cortical thickness (B), and surface area (C) as exemplars. Within each panel, the left-hand figure shows the Z-scores of each participant at baseline and follow-up and the right-hand figure shows the distribution of the MAE and RMSE at baseline and follow-up. Note that scales on x axes differ between plots. MAE=mean absolute error. MFPR=multivariate fractional polynomial regression. RMSE=root mean square error. SLIM=Southwest Longitudinal Imaging Multimodal Study. QTAB= Queensland Twin Adolescent Brain Study.

For an optimised MFPR model performance in longitudinal datasets we show the stability of the regional Z-scores derived with the optimised MFPR models applied to structural MRI data of healthy participants in the SLIM and QTAB samples scanned with an average interval of approximately 2 years (figure 7).

**Figure 8:**
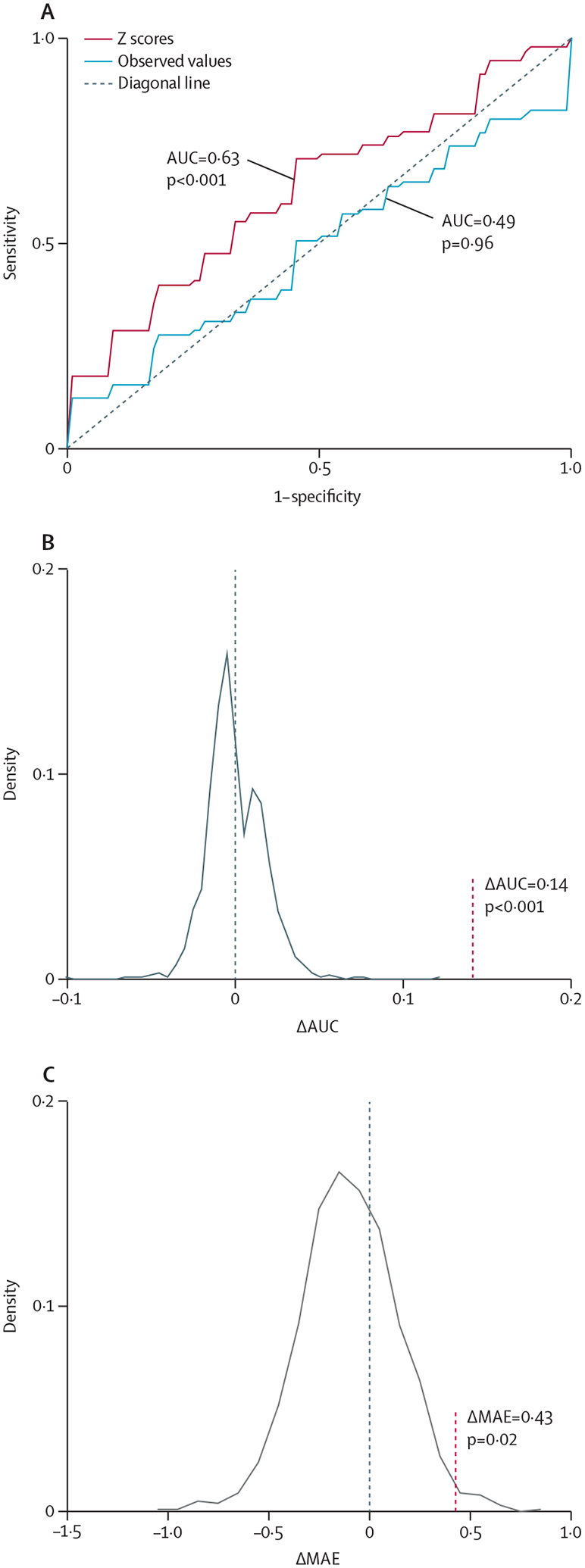
Accuracy of diagnostic classification and accuracy of psychotic symptom prediction using brain regional normative deviation scores or observed neuromorphometric data The diagnostic classification accuracy in the HCP-EP sample (A): receiver operating characteristic curves of the models distinguishing patients from controls with the observed regional neuromorphometric measures (blue curve) or the deviation Z-scores from the normative model (red curve); the AUC difference between a support vector machine classifier using the observed regional neuromorphometric measures and another using regional normative deviation scores (Z-scores) derived from the optimised MFPR model was examined through 1000 permutations (B): the AUC difference is marked by a vertical dotted line; the predictive accuracy of psychotic symptoms in the HCP-EP sample (C): the MAE difference between a ridge regression using the observed regional neuromorphometric measures and another using Z-scores derived from the optimised MFPR model was examined through 1000 permutations, the MAE difference is marked by a vertical dotted line. Information on other models is provided in appendix 1 (p 22). Note that scales on axes differ between plots. AUC=area under the curve. HCP-EP=Human Connectome Project-Early Psychosis. MFPR=multivariate fractional polynomial regression.

In the HCP-EP cohort, the accuracy of the diagnostic classification of the SVCs that used regional Z-scores performed similarly regardless of the normative model and outperformed the SVC with the observed data. In figure 8, we illustrate these findings by showing that the SVC that used Z-scores from the optimised MFPR models achieved an AUC of 0·63 (p<0·001) whereas the accuracy of the SVC that used observed data was indistinguishable from chance (AUC 0·49). Information on other models is in appendix 1 (p 22).

The predictive accuracy for psychotic symptom severity of the ridge regression models using the Z-scores from the different normative models performed similarly to each other and to the model using observed data; none achieved an above chance performance (appendix 1 p 22). In figure 8, we illustrate these findings by showing the predictive accuracy of the regression models using optimised MFPR-derived Z-scores or observed data.

## Discussion

This study undertook a comparative evaluation of eight algorithms commonly used for normative modelling using morphometric data from a multisite sample of 37 407 healthy individuals. Sex-specific models based on the MFPR algorithm with non-linear fractional polynomials of age and linear global neuroimaging measures emerged as optimum based on their performance and computational efficiency, with computational efficiency being an important consideration when analysing large datasets. These models were robust to variations in sample composition with respect to age and their performance plateaued at sample sizes of approximately 3000 people. The optimised sex-specific MFPR models showed longitudinal stability over an average interval of 2 years and the Z-scores derived from these models outperformed observed neuromophometric measures in distinguishing patients with psychosis from healthy individuals.

The findings validate our choice to use MFPR in our previous normative studies on brain morphometry^2,3^ and white matter microstructure based on diffusion-weighted MRI.^25^ Furthermore, after testing the effect of multiple combinations of explanatory variables on model performance, we found that global morphometric measures (ie, intracranial volume, mean cortical thickness, and total cortical surface area) had the greatest significant effect. This observation is aligned with previous literature on the contribution of intracranial volume in explaining the variance of regional subcortical volumes and cortical surface area measures.^26,27^ This study extended these findings by showing that mean cortical thickness and mean surface area outperformed intracranial volume as explanatory variables in normative models of regional cortical thickness and cortical surface area. Accordingly, the optimal normative models for brain morphometry consisted of an MFPR algorithm and a combination of explanatory variables that comprised non-linear fractional polynomials of age and linear global measures of intracranial volume (for models of regional subcortical volume), mean cortical thickness (for models of regional cortical thickness), and mean cortical surface area (for models of regional cortical surface area). Sensitivity analyses across different age bins supported the applicability of the models developed in the whole study sample, which spanned an age range of 3–90 years, to groups with a more restricted age range and at different points in their life trajectories. The optimised sex-specific MFPR models showed longitudinal stability over an average follow-up period of 2 years as would be expected for healthy adults over short time periods.^1–3^

Site variation is a major challenge when aggregating multisite data as it can confound or bias results. The most common methods for minimising site effects involve either site harmonisation using ComBat-GAM before normative modelling or the inclusion of site as an explanatory variable in the normative models. One publication that used a smaller sample (569 healthy participants) and a narrower age range (6–40 years) suggested that HBR with site as an explanatory variable might be superior to ComBat-based site harmonisation for the normative modelling of brain morphometry.^12^ We found no support for this assertion in our sensitivity analyses. An additional advantage of using ComBat-GAM is that it removes the requirement for calibration and model parameter adaptation every time the model is applied to data from a new site. By contrast, in the HBR models, pretrained parameters can be used for new data if they originate from one of the sites in the training dataset^10^ or under the assumption that the variation accounted for by an unseen site should align with that of the sites in the training dataset.^12^

Previous studies have shown that sex accounts for a considerable amount of variance in brain morphology, both cross-sectionally^16^ and longitudinally.^28^ Accordingly, we developed sex-specific models for each brain morphometric measure, thus extending previous normative studies that considered males and females together.^1,13^ Additionally, we provide normative models for regional cortical surface area measures that were not included in previous studies^1,13^ despite the important functional implications of age-related changes in the cortical surface area for cognition during development and ageing. We note that the current normative model is compiled cross-sectionally, from people of different ages who had different exposures to factors that can influence brain health. In later life, samples of healthy individuals are likely to include those who are more resilient to mortality and morbidity.

There are several methodological limitations pertinent to this study. Specifically, our study could benefit from the inclusion of more young and middle-aged adults and data from longitudinal follow-up over long periods of time. Testing the generalisability of our models to populations with specific ancestries is an important next step. We did not include an exhaustive list of potential explanatory variables. It could be argued that the inclusion of other variables, such as childhood adversity, premature birth, or socioeconomic status, which are known to influence brain morphometry,^29,30^ could have further improved model performance. Exploring this possibility further could be best achieved within the context of single large-scale studies in which such variables would be consistently recorded in all participants. On the other hand, the inclusion of multiple explanatory variables in the normative model itself could restrict its applicability to only those datasets in which all such features were assessed.

In conclusion, this study presents a detailed evaluation of the comparative performance of the key eight algorithms used for normative modelling and of the influence of key parameters pertaining to site effects, covariates, sample size, and sample composition with respect to age on model accuracy and robustness. On the basis of the evidence provided, we consider the sex-specific optimised MFPR models developed here to be advantageous in terms of accuracy and efficiency compared with other options. We therefore provide these models in CentileBrain, a user-friendly web platform that enables the estimation of normative deviation scores from any sample with minimal technical and computing requirements.

## Supplementary Material

1

2

3

4

5

6

## Data Availability

Access to individual participant data from each dataset is available through access requests addressed to the principal investigators of the original studies or to the relevant data repositories. Details are provided in appendix 2. A dedicated web portal (https://centilebrain.org) provides the optimal model parameters, as pretrained models, to be applied to any user-specified dataset in the context of open science.

## Search strategy and selection criteria

Normative reference values of neuroimaging measures of brain structure and function have great potential as clinical and research tools, but the models used to generate these values must be methodologically robust. We searched electronic databases for articles published in English between Jan 1, 2018, and Jan 31, 2023, with combinations of words or terms that included “normative modeling”, OR “growth curves” OR “centile curves” AND terms referring to specific morphometric features (ie, cortical thickness, cortical surface area, and subcortical volume).
