# Supplementary material for: Normative modelling of brain morphometry across the lifespan with CentileBrain: algorithm benchmarking and model optimisation
Source: Lancet Digit Health. Author manuscript; Available in PMC 2024 Mar 12. (PMC10929064; doi:10.1016/S2589-7500(23)00250-9)
Supplement: 1 [file NIHMS1969418-supplement-1.pdf]

THE LANCET
Digital Health

Supplementary appendix

This appendix formed part of the original submission and has been peer reviewed. We post it as supplied by the authors.

Supplement to: Ge R, Yu Y, Qi YX, et al. Normative modelling of brain morphometry across the lifespan with CentileBrain: algorithm benchmarking and model optimisation. *Lancet Digit Health* 2024; **6:** e211–21.

Normative Modeling of Brain Morphometry Across the Lifespan Using CentileBrain: Algorithm Benchmarking and Model Optimization
Supplementary Material

Appendix 1: Table of Contents

S1. Samples **2**
Supplementary Figure S1. Age-by-sex distribution of the study sample 2
S2. Quality assessment of neuroimaging data **3**
Supplementary Figure S2. Illustrative results of the minimal effect of Euler Number on the performance of the Multivariate Fractional Polynomial Regression models. 3
S3. Comparative evaluation of algorithms **4**
Supplementary Figure S3. Illustrative example of comparative algorithm performance for males 4
Supplementary Figure S4. Comparative algorithm performance of region-specific models in females 5
Supplementary Figure S5. Comparative algorithm performance of region-specific models in males 6
S4. Selection of explanatory variables for model optimization **7**
Supplementary Figure S6. Illustrative examples of the performance of MFPR-derived models as a function of explanatory variables for males 7
Supplementary Figure S7. Performance of region-specific MFPR-derived models as a function of explanatory variables in females 8
Supplementary Figure S8. Performance of region-specific MFPR-derived models as a function of explanatory variables in males 9
Supplementary Figure S9. Illustrative examples of the comparative performance of HBR, OLSR, BLR, GAMLSS, GPR, WBLR, and MFPR-derived models in males 10
Supplementary Figure S10. Comparative performance of all region-specific optimized HBR, OLSR, BLR, GAMLSS, GPR, WBLR, and MFPR-derived models in females 11
Supplementary Figure S11. Comparative performance of all region-specific optimized HBR, OLSR, BLR, GAMLSS, GPR, WBLR, and MFPR-derived models in males 12
S5. Sensitivity analyses **13**
S5.1 Sample size **13**
Supplementary Figure S12. Performance of region-specific MFPR-derived models as a function of sample size for males 13
S5.2 Age bins **14**
Supplementary Figure S13. Performance of region-specific models in distinct age groups for males 14
S5.3 Sensitivity analyses pertaining to the GAMLSS **16**
Supplementary Figure S14. Comparative evaluations of five GAMLSS models in females 17
Supplementary Figure S15. Comparative evaluations of five GAMLSS models in males 18
Supplementary Figure S16. Comparative evaluations of GAMLSS models using the “caret” and “gamlss” software in females 19
Supplementary Figure S17. Comparative evaluations of GAMLSS models using the “caret” and “gamlss” software in males 20
S5.4 Sensitivity analyses pertaining to site **21**
Supplementary Figure S18. Effect of site handling on algorithm performance 21
S6. Clinical relevance of normative brain models for mental illness **22**
Supplementary Figure S19. Comparative accuracy of diagnostic classification and symptom severity prediction in psychosis using either brain regional Z-scores or observed neuromorphometric data of the Human Connectome Project-Early Psychosis cohort 22
S7. List of appendices 2-6 in Excel format **23**
Supplementary references **24**
ENIGMA Lifespan working group authors and affiliations **25**

S1. Samples

We collated data from 87 datasets. The age-by-sex distribution of the current study sample is shown in supplementary figure S1 and details of the contributing datasets are provided in appendix 2. In the Figure below, we also illustrate the geographic distribution of the sample, with different colors representing the geographical region of each site. The clustering was conducted on all 150 morphometric measures (68 cortical thickness measure, 68 surface area measures, and 14 subcortical volume measures) of the entire sample. We did not identify region-specific clusters. The Figure was generated using t-SNE (t-distributed stochastic neighbor embedding) plot to visualize the high dimensional data into a 2D plot.

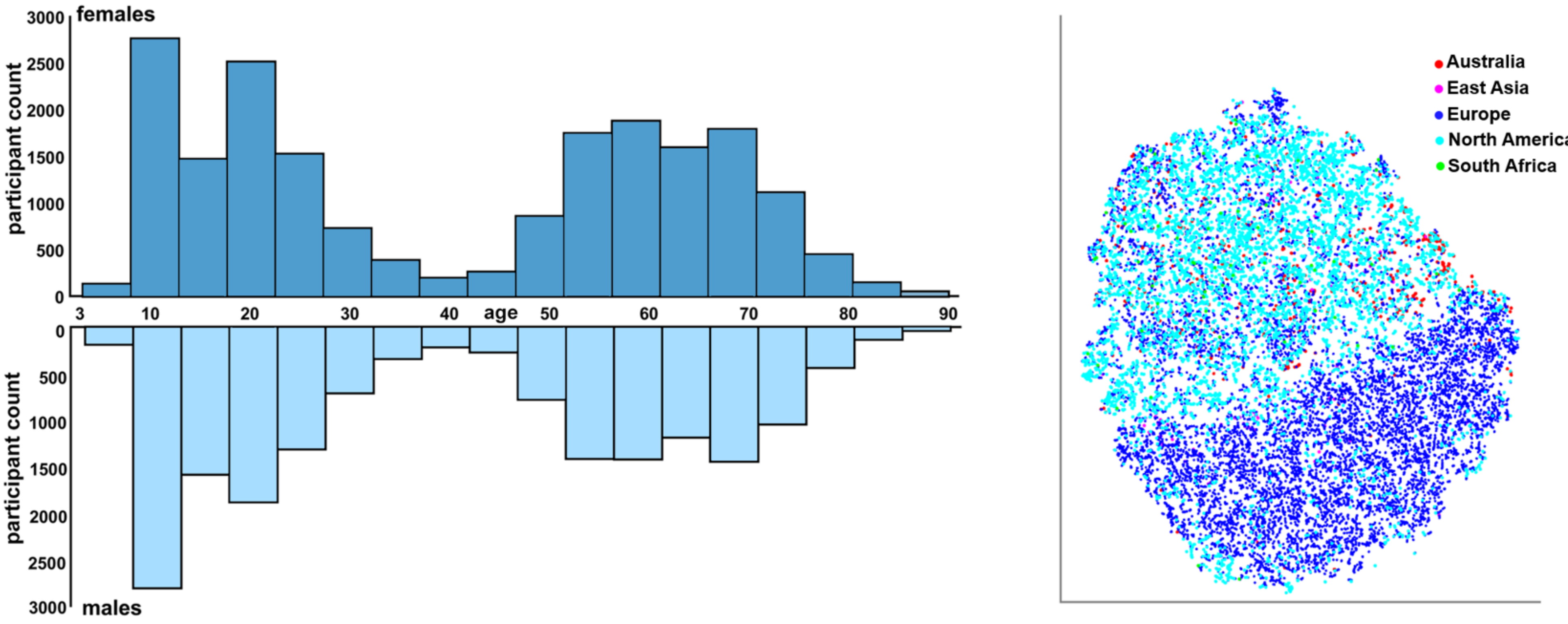

Supplementary Figure S1. (left panel) Age-by-sex distribution of the study sample, and (right panel) geographic distribution of the sample.

S2. Quality assessment of neuroimaging data

For samples participating in the ENIGMA Lifespan Working Group (appendix 2), quality assessment followed the ENIGMA consortium pipeline (http://enigma.ini.usc.edu/protocols/imaging-protocols/).[1–7] These pipelines were therefore applied to 18,787 individuals in the study sample. Data from the Adolescent Brain Cognitive Development study were considered high-quality based on their quality assessment protocols (Txt file= FreeSurfer QC; item= fsqc_qc; scan excluded if 0).[8] This applied to 3,116 individuals in the study sample. For the remainder of the sample involving 15,504 participants, the T1-weighted images were downloaded and segmented locally, and the results were quality assessed using Qoala-T tool.[9]

As an additional assessment of image quality, we calculated the Euler number (EN), a reliable indicator that closely approximates the manual assessment of scan quality[10, 11] for the entire dataset. The addition of the EN to the covariates did not make a measurable difference in any model. We illustrate that for the Multivariate Fractional Polynomial Regression (MFPR) models for the left thalamic volume in males using the UKB data which are publicly accessible. The pattern observed for all other regions and models in each sex is the same and the data are available upon request.

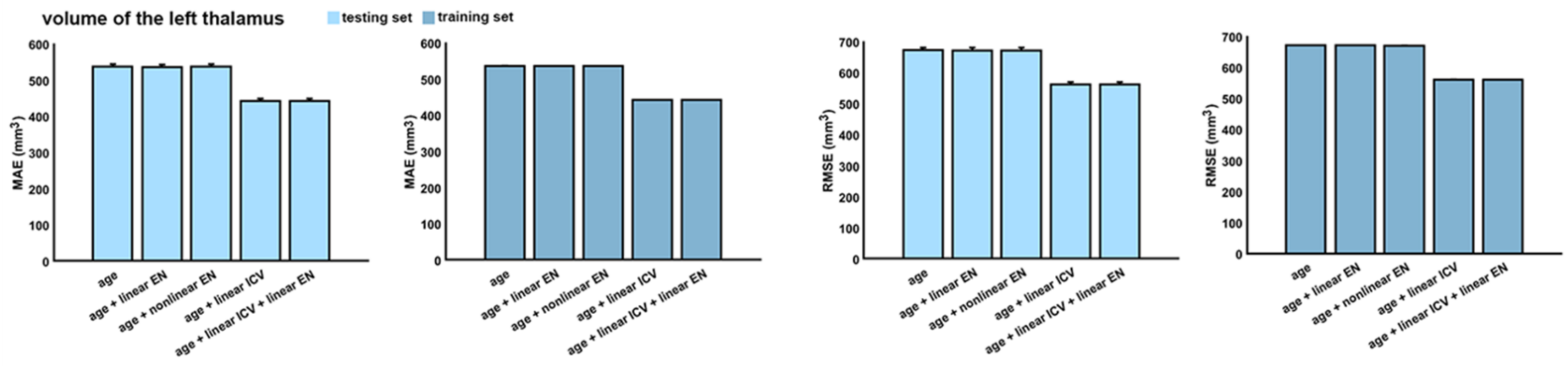

Supplementary Figure S2. Illustrative results of the minimal effect of Euler Number on the performance (MAE; mean absolute error and RMSE; root mean square error) of the Multivariate Fractional Polynomial Regression models.

S3. Comparative evaluation of algorithms

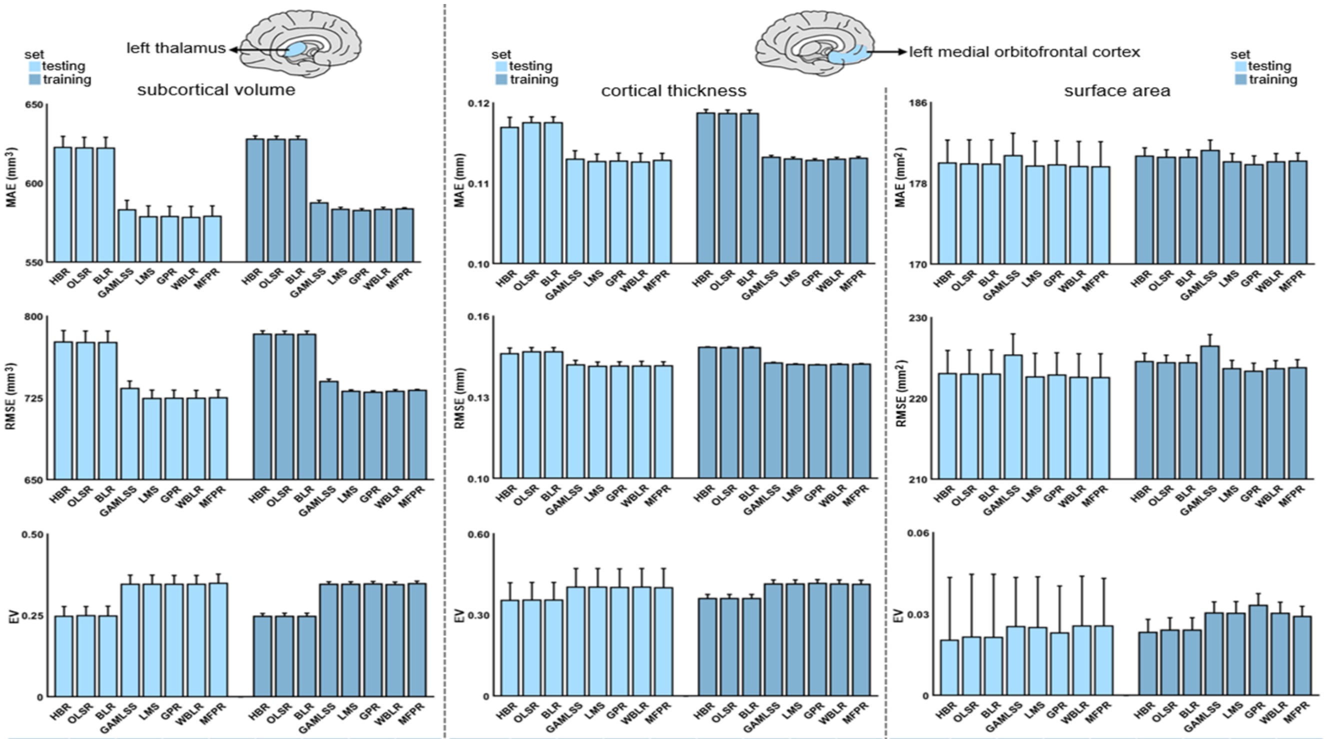

algorithm	HBR	OLSR	BLR	GAMLSS	LMS	GPR	WBLR	MFPR
CPU time (seconds)	651.29 ±140.58	0.01 ±0.01	5.82 ±0.37	3.16 ±0.06	3.85 ±0.10	1814.16 ±8.27	0.88 ±0.07	0.42 ±0.04

algorithm	HBR	OLSR	BLR	GAMLSS	LMS	GPR	WBLR	MFPR
CPU time (seconds)	493.18 ±236.18	0.01 ±0.001	4.43 ±0.22	1.92 ±0.24	2.71 ±0.02	1651.53 ±45.08	0.82 ±0.04	0.34 ±0.03

algorithm	HBR	OLSR	BLR	GAMLSS	LMS	GPR	WBLR	MFPR
CPU time (seconds)	128.81 ±14.02	0.06 ±0.13	5.02 ±0.98	3.69 ±0.29	2.76 ±0.41	1779.09 ±81.07	0.79 ±0.04	0.40 ±0.07

Supplementary Figure S3. Illustrative example of comparative algorithm performance for males. Algorithm performance for each regional morphometric measure was assessed separately in males and females using the mean absolute error (MAE), the root mean square error (RMSE), the Explained Variance (EV), and the central processing unit (CPU) time. The MAE, RMSE, EV, and CPU times of the models for left thalamic volume (left panel), the left medial orbitofrontal cortical thickness (middle panel), and surface area (right panel) as exemplars are presented here for males and in figure 2 in the main text for females. HBR=Hierarchical Bayesian Regression; OLSR=Ordinary Least Squares Regression; BLR=Bayesian Linear Regression; GAMLSS=Generalized Additive Models for Location, Scale, and Shape; LMS=Lambda (λ), Mu (μ), Sigma (σ) Method; GPR=Gaussian Process Regression; WBLR=Warped Bayesian Linear Regression; MFPR=Multivariable Fractional Polynomial Regression.

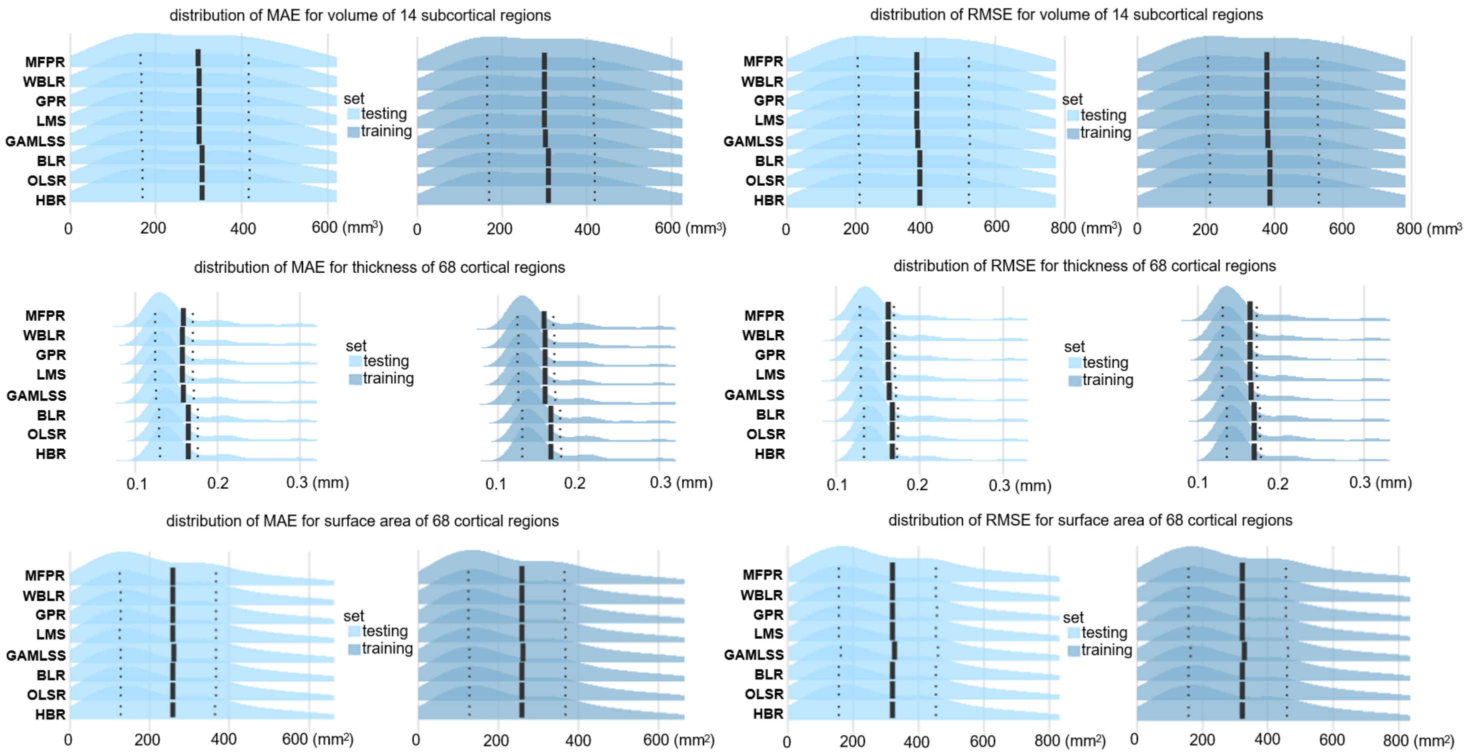

Supplementary Figure S4. Comparative algorithm performance of region-specific models in females. The figure presents the distribution of the mean absolute error (MAE) and the root mean square error (RMSE) of all region-specific models. Mean MAE and RMSE values are marked with solid vertical lines, and the 25th percentiles and 75th percentiles are marked with dotted vertical lines. HBR=Hierarchical Bayesian Regression; OLSR=Ordinary Least Squares Regression; BLR=Bayesian Linear Regression; GAMLSS=Generalized Additive Models for Location, Scale, and Shape; LMS=Lambda (λ), Mu (μ), Sigma (σ) Method; GPR=Gaussian Process Regression; WBLR=Warped Bayesian Linear Regression; MFPR=Multivariable Fractional Polynomial Regression.

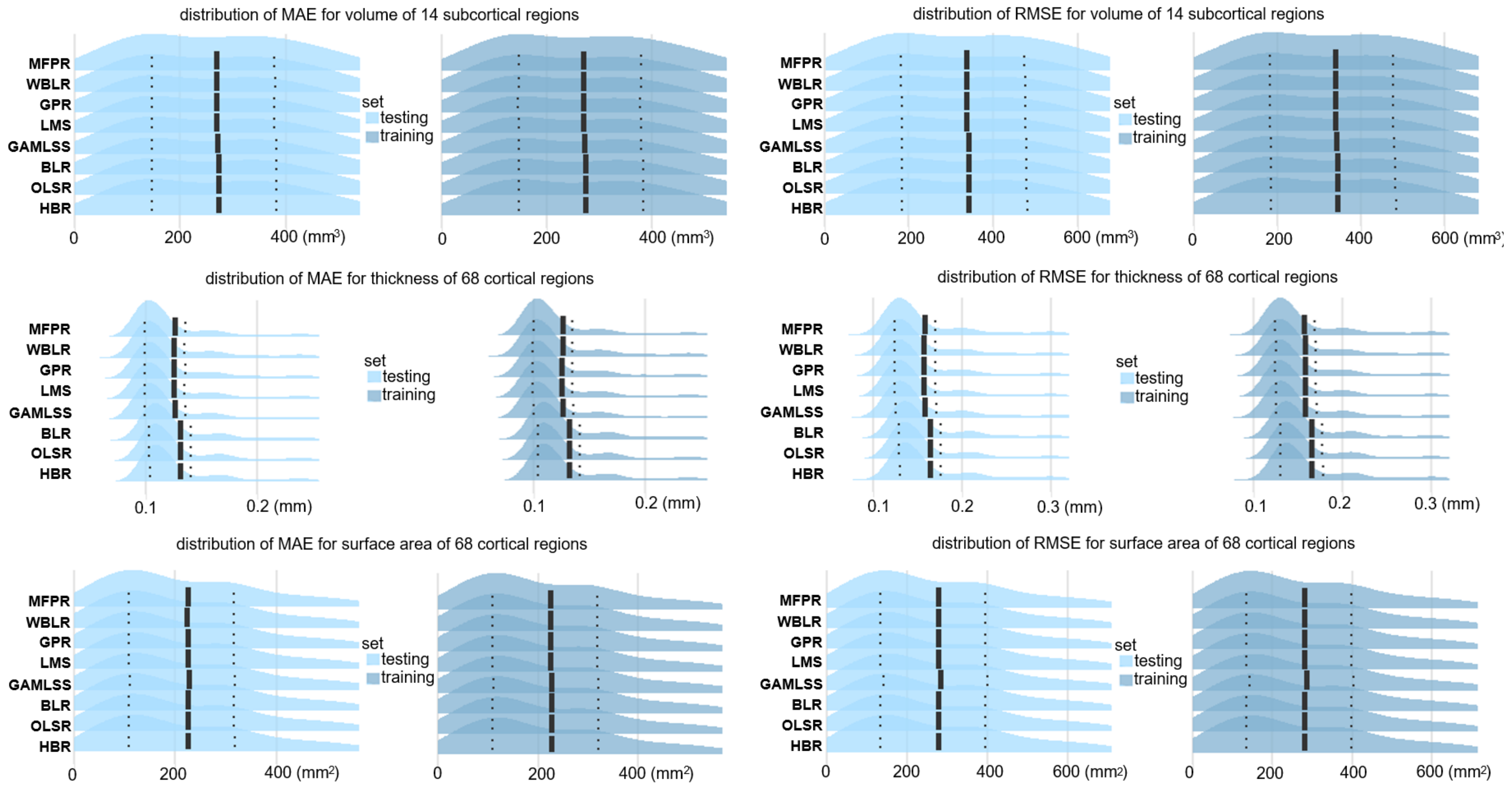

Supplementary Figure S5. Comparative algorithm performance of region-specific models in males. The figure presents the distribution of the mean absolute error (MAE) and the root mean square error (RMSE) of all region-specific models. Mean MAE and RMSE values are marked with solid vertical lines, and the 25th percentiles and 75th percentiles are marked with dotted vertical lines. HBR=Hierarchical Bayesian Regression; OLSR=Ordinary Least Squares Regression; BLR=Bayesian Linear Regression; GAMLSS=Generalized Additive Models for Location, Scale, and Shape; LMS=Lambda (λ), Mu (μ), Sigma (σ) Method; GPR=Gaussian Process Regression; WBLR=Warped Bayesian Linear Regression; MFPR=Multivariable Fractional Polynomial Regression.

S4. Selection of explanatory variables for model optimization

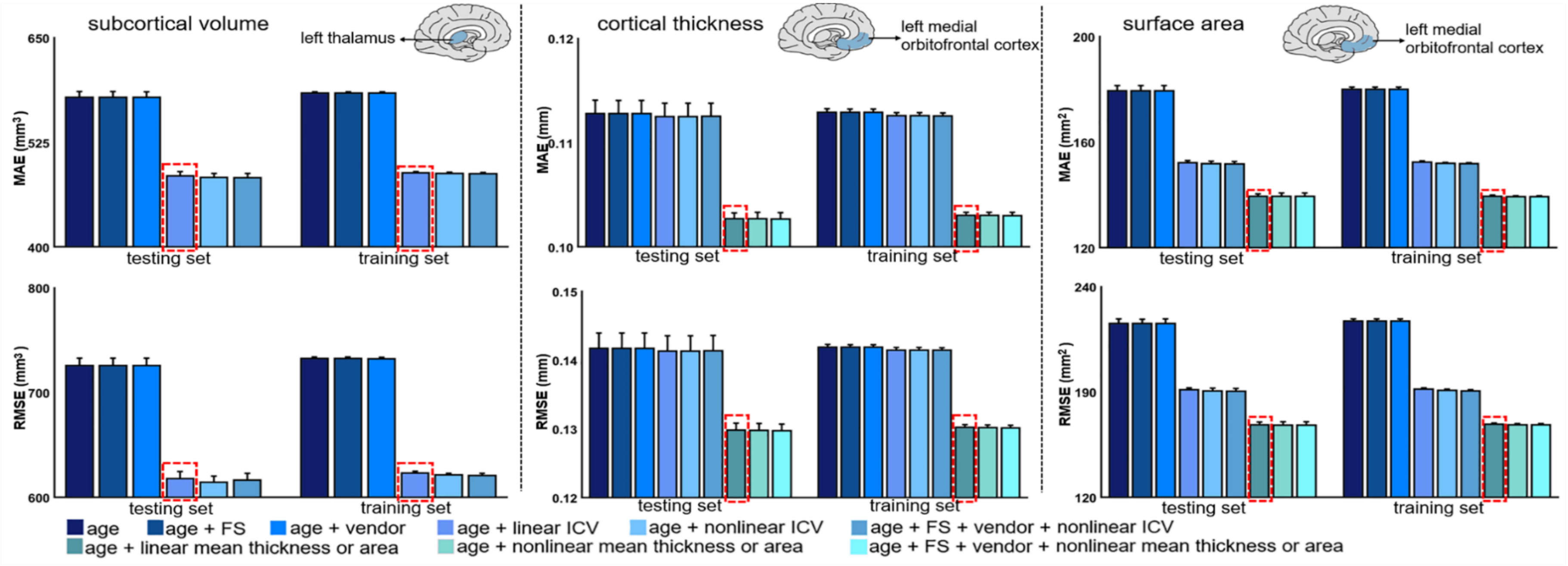

Supplementary Figure S6. Illustrative examples of the performance of MFPR-derived models as a function of explanatory variables for males. For each regional morphometric measure, sex-specific models derived from Multivariable Fractional Polynomial Regression (MFPR) were trained and tested using nine different covariate combinations that included linear and non-linear effects of age, FreeSurfer version (FS), scanner vendor, intracranial volume (ICV), and global estimates of mean cortical thickness or area. The mean absolute error (MAE) and root mean square error (RMSE) of all the models in males and females are shown in supplementary figures S7–S8 and appendix 4. In both sexes, the pattern identified was identical for all region-specific models. The MAE and RMSE of the models for left thalamic volume (left panel), the left medial orbitofrontal cortical thickness (middle panel) and surface area (right panel) as exemplars are presented here for males and in figure 3 in the main text for females. The optimal variable combination is marked with a dashed frame.

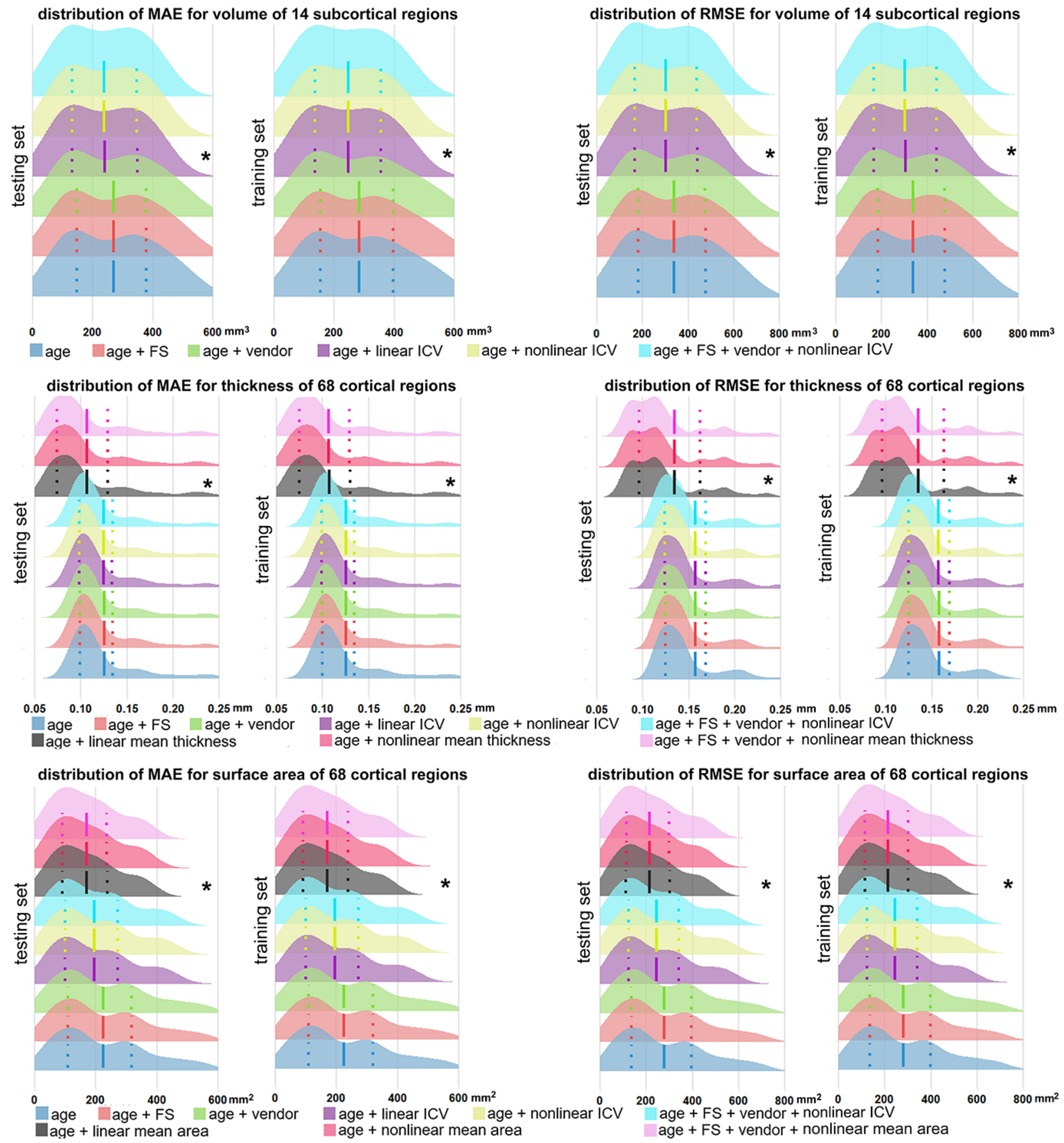

Supplementary Figure S7. Performance of region-specific MFPR-derived models as a function of explanatory variables in females. The figure presents the distribution of the mean absolute error (MAE) and the root mean square error (RMSE) for all region-specific models derived from Multivariable Fractional Polynomial Regression (MFPR) as a function of explanatory variables in females. The mean MAE and RMSE values are marked with solid vertical lines, and the 25th percentiles and 75th percentiles are marked with dotted vertical lines. The optimal variable combination is marked with an asterisk. FS=FreeSurfer version; ICV=intracranial volume.

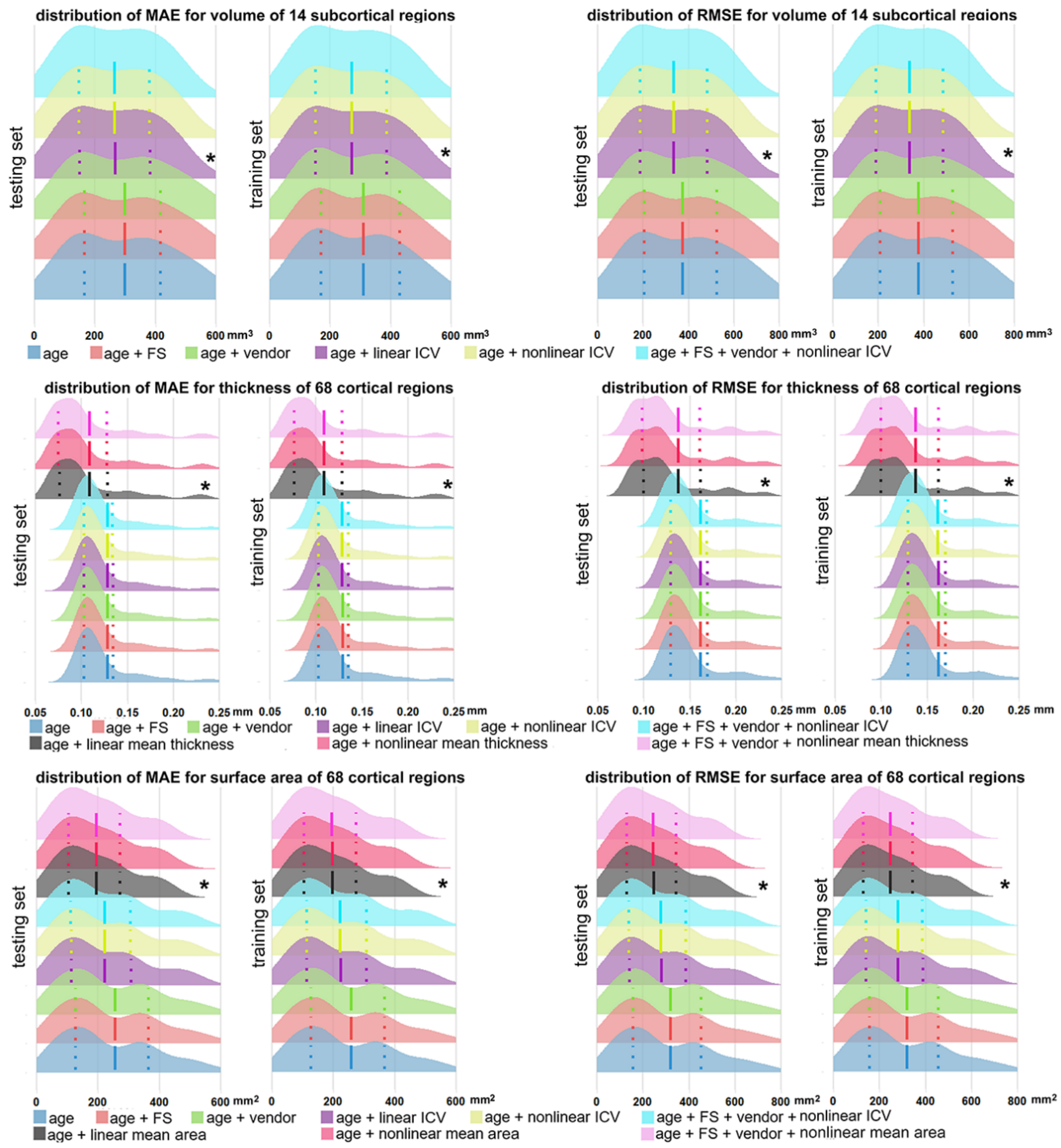

Supplementary Figure S8. Performance of region-specific MFPR-derived models as a function of explanatory variables in males. The figure presents the distribution of the mean absolute error (MAE) and the root mean square error (RMSE) for all region-specific models derived from Multivariable Fractional Polynomial Regression (MFPR) as a function of explanatory variables in males. The mean MAE and RMSE values are marked with solid vertical lines, and the 25th percentiles and 75th percentiles are marked with dotted vertical lines. The optimal variable combination is marked with an asterisk. FS=FreeSurfer version; ICV=intracranial volume.

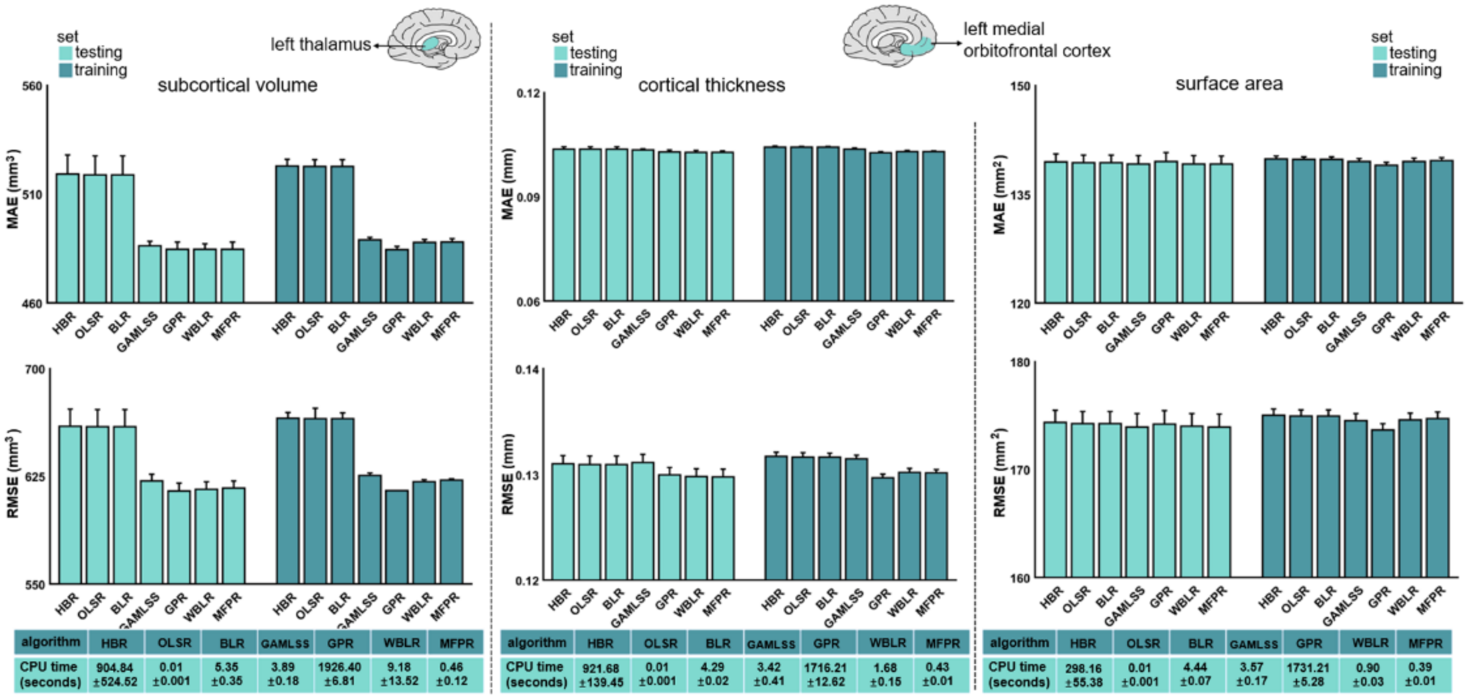

algorithm	HBR	OLSR	BLR	GAMLSS	GPR	WBLR	MFPR
CPU time (seconds)	904.84 ±524.52	0.01 ±0.001	5.35 ±0.35	3.89 ±0.18	1926.40 ±6.81	9.18 ±13.52	0.46 ±0.12

algorithm	HBR	OLSR	BLR	GAMLSS	GPR	WBLR	MFPR
CPU time (seconds)	921.68 ±139.45	0.01 ±0.001	4.29 ±0.02	3.42 ±0.41	1716.21 ±12.62	1.68 ±0.15	0.43 ±0.01

algorithm	HBR	OLSR	BLR	GAMLSS	GPR	WBLR	MFPR
CPU time (seconds)	298.16 ±55.38	0.01 ±0.001	4.44 ±0.07	3.57 ±0.17	1731.21 ±5.28	0.90 ±0.03	0.39 ±0.01

Supplementary Figure S9. Illustrative examples of the comparative performance of HBR, OLSR, BLR, GAMLSS, GPR, WBLR, and MFPR-derived models in males. Region-specific models with the optimized covariate combination were estimated in males and females separately using Hierarchical Bayesian Regression (HBR), Ordinary Least Squares Regression (OLSR), Bayesian Linear Regression (BLR), Generalized Additive Models for Location, Scale, and Shape (GAMLSS), Gaussian Process Regression (GPR), Warped Bayesian Linear Regression (WBLR), and Multivariable Fractional Polynomial Regression (MFPR). Model performance was assessed in terms of mean absolute error (MAE), root mean square error (RMSE), and central processing unit (CPU). The MAE, RMSE, and CPU time of the models for left thalamic volume (left panel), the left medial orbitofrontal cortical thickness (middle panel), and surface area (right panel) as exemplars are presented here for males and in figure 4 in the main text for females.

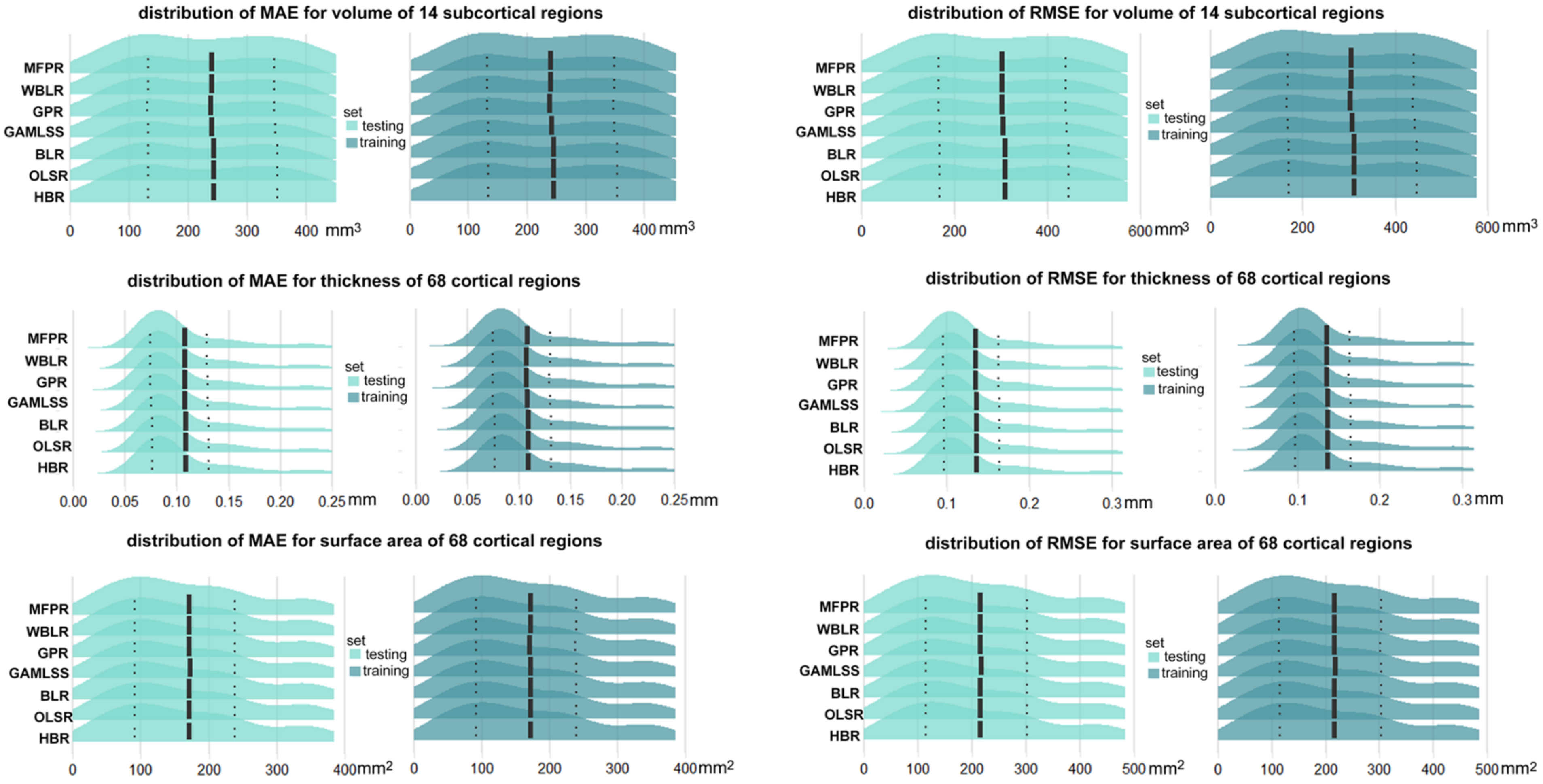

Supplementary Figure S10. Comparative performance of all region-specific optimized HBR, OLSR, BLR, GAMLSS, GPR, HBR, WBLR, and MFPR-derived models in females. The figure presents the distribution of the mean absolute error (MAE) and the root mean square error (RMSE) for all region-specific models derived from Hierarchical Bayesian Regression (HBR), Ordinary Least Squares Regression (OLSR), Bayesian Linear Regression (BLR), Generalized Additive Models for Location, Scale, and Shape (GAMLSS), Gaussian Process Regression (GPR), Warped Bayesian Linear Regression (WBLR), and Multivariable Fractional Polynomial Regression (MFPR). The mean MAE and RMSE values are marked with solid vertical lines, and the 25th percentiles and 75th percentiles are marked with dotted vertical lines.

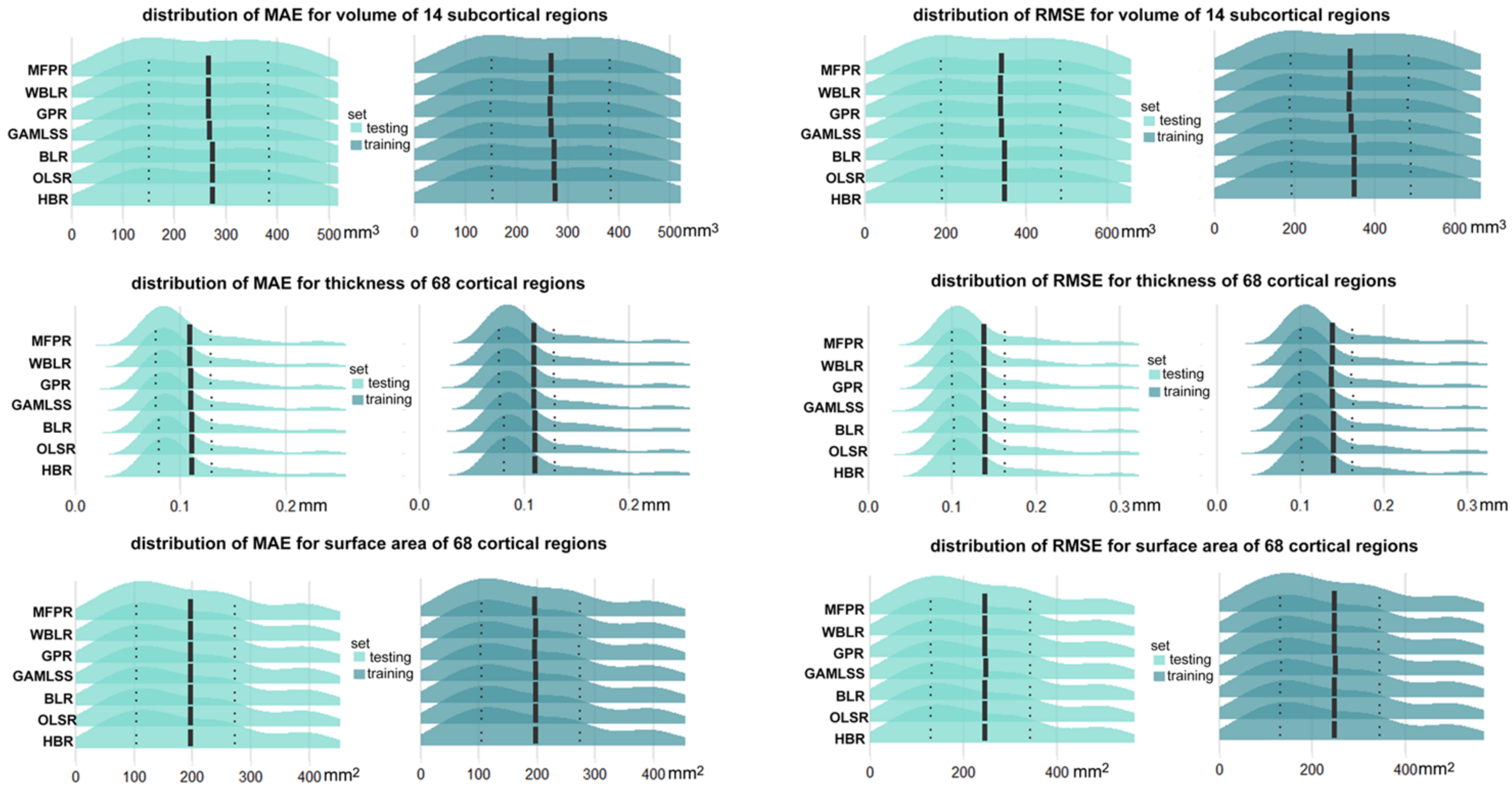

Supplementary Figure S11. Comparative performance of all region-specific optimized HBR, OLSR, BLR, GAMLSS, GPR, HBR, WBLR, and MFPR-derived models in males. The figure presents the distribution of the mean absolute error (MAE) and the root mean square error (RMSE) for all region-specific models derived from Hierarchical Bayesian Regression (HBR), Ordinary Least Squares Regression (OLSR), Bayesian Linear Regression (BLR), Generalized Additive Models for Location, Scale, and Shape (GAMLSS), Gaussian Process Regression (GPR), Warped Bayesian Linear Regression (WBLR), and Multivariable Fractional Polynomial Regression (MFPR). The mean MAE and RMSE values are marked with solid vertical lines, and the 25th percentiles and 75th percentiles are marked with dotted vertical lines.

S5. Sensitivity analyses
S5.1 Sample size

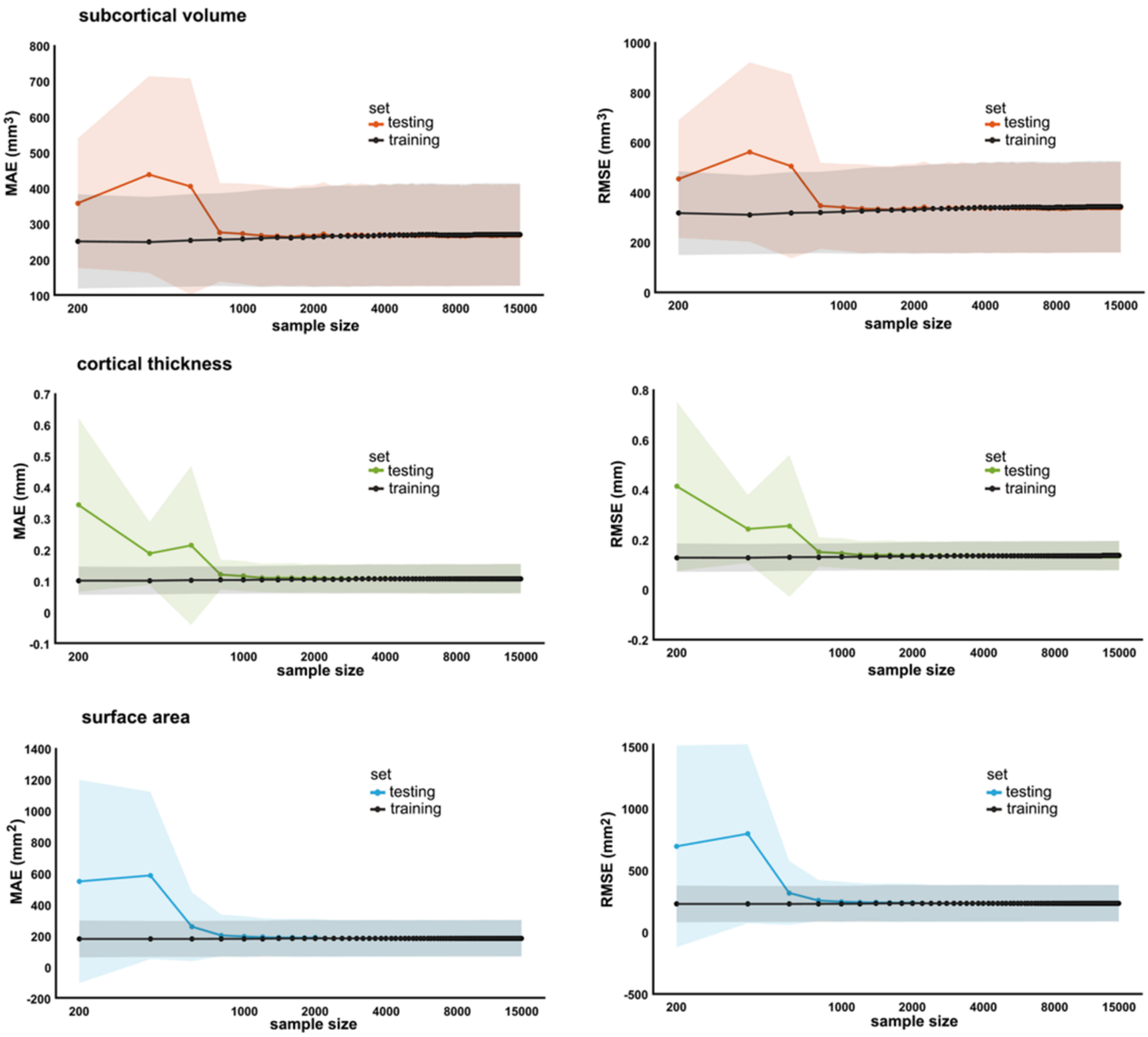

Supplementary Figure S12. Performance of region-specific MFPR-derived models as a function of sample size for males. Models for each regional morphometric measure were estimated in random sex-specific subsets of 200 to 15,000 participants, in increments of 200, generated from the study sample. Each line represents the values of the mean absolute error (MAE), or root mean square error (RMSE) derived from the optimized Multivariable Fractional Polynomial Regression (MFPR) models of each regional morphometric measure as a function of sample size. The pattern identified was identical in both sexes. The data for males are shown here and in figure 5 in the main text for females.

S5.2 Age bins

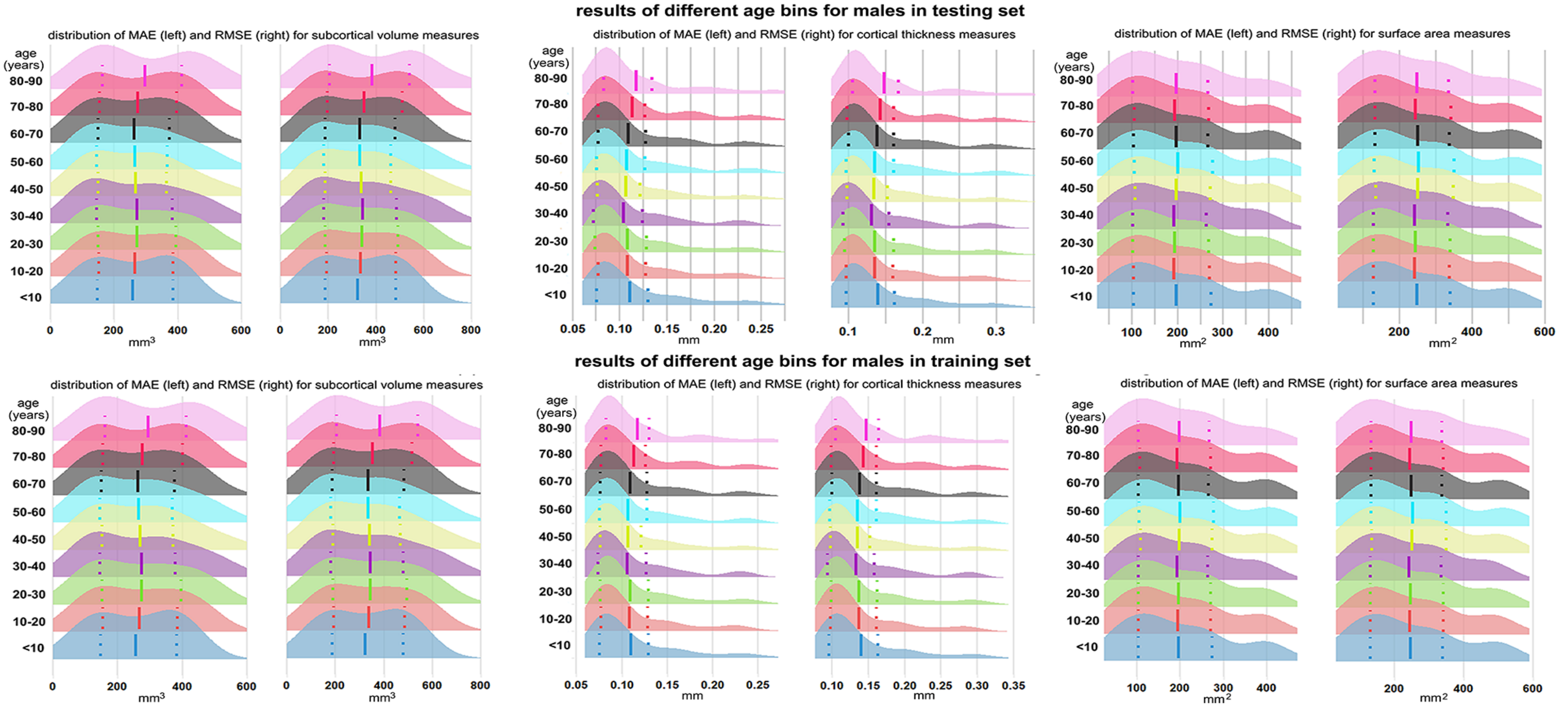

Supplementary Figure S13. Performance of region-specific models in distinct age groups for males. Sex- and region-specific models for all morphometric measures for different age groups were estimated by partitioning the sex-specific training and testing subsets of the study sample into nine age bins (i.e., age≤10 years; 10<age≤20 years; 20<age≤30 years; 30<age≤40 years; 40<age≤50 years; 50<age≤60 years; 60<age≤70 years; 70<age≤80 years; 80<age≤90 years). Details are provided in appendix 6. The pattern was identical in both sexes. Supplementary figure S9 presents the distribution of the mean absolute error (MAE) and the root mean square error (RMSE) across all region-specific models in males in the training (upper panel) and test subset (lower panel). The results for females are presented in figure 6 in the main text.

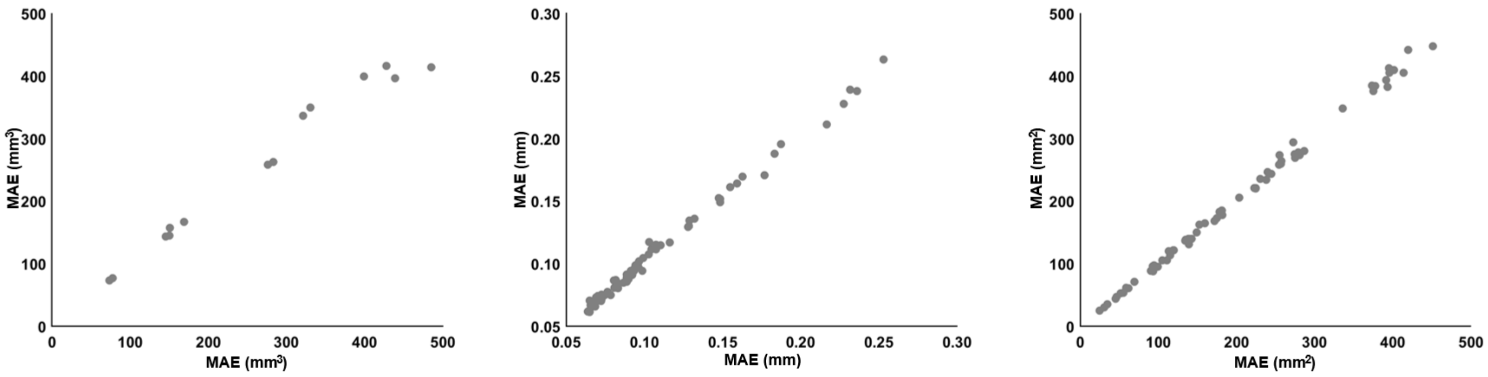

Supplementary Figure S13 (continued). Scatter plots between the MAE values of the models within the first age bin (age≤10 years) of male participants with those derived from the entire male sample. Across all age bins, the correlation coefficient between the MAE or RMSE values of the sex- and region-specific models obtained from the full study sample and MAE or RMSE values of the corresponding models estimated in each age bin were all greater than 0·98. Same pattern was observed for all age bins of both sexes.

S5. 3 Sensitivity analyses pertaining to the GAMLSS

Prior studies have focused on GAMLSS algorithms and their relative performance in normative modeling of brain morphometric data. A key example is the paper by Dinga and colleagues.[12] As shown in supplementary figures S14 and S15 for females and males below, five models were compared: Model 1 is a Gaussian model with linear effect; Model 2 is a non-linear Gaussian homoscedastic model; Model 3 is a non-linear Gaussian heteroscedastic model; Model 4 is a non-linear heteroscedastic SHASH model; and Model 5 is a non-linear heteroscedastic SHASH model where location, scale, and shape also depend on age.

Model 1: regional neuromorphometric measure ~ $N(\mu, \sigma), \mu = \beta_0 + \beta_{age} * age$

Model 2: regional neuromorphometric measure ~ $N(\mu, \sigma), \mu = f_\mu(age)$

Model 3: regional neuromorphometric measure ~ $N(\mu, \sigma), \mu = f_\mu(age), \sigma = f_\sigma(age)$

Model 4: regional neuromorphometric measure ~ $SHASH(\mu, \sigma, \nu, \tau), \mu = f_\mu(age), \sigma = f_\sigma(age), \nu = \beta_{nu}, \tau = \beta_{tau}$

Model 5: regional neuromorphometric measure ~ $SHASH(\mu, \sigma, \nu, \tau), \mu = f_\mu(age), \sigma = f_\sigma(age), \nu = f_\nu(age),\ \tau = f_\tau(age)$

Model 4 is the model used in our manuscript. All other models are from Dinga et al.[12] These five GAMLSS models were compared in terms of MAE (mean absolute error), kurtosis, and skewness. All models have similar MAE, while model 4 and model 5 performed best in terms of kurtosis and skewness (i.e., for kurtosis and skewness, closer to 0 is better). Since differences in the performance of models 4 and 5 are negligible, the results support our choice of using model 4, which is also simpler (figure 2 in the main text). The same pattern was found for RMSE (root mean squared error) and EV (explained variance).

We also compared the performance of the GAMLSS models using either the "caret" or "gamlss" packages in terms of MAE, kurtosis, and skewness using the same sample as above. The results shown in supplementary figures S14-S15 demonstrate the similarity between the two packages in MAE. The output of the "caret" package used in our paper performed better in terms of the model fit of kurtosis and skewness (for kurtosis and skewness, closer to 0 is better) for the testing set. These analyses all support the choice of the package used in our manuscript.

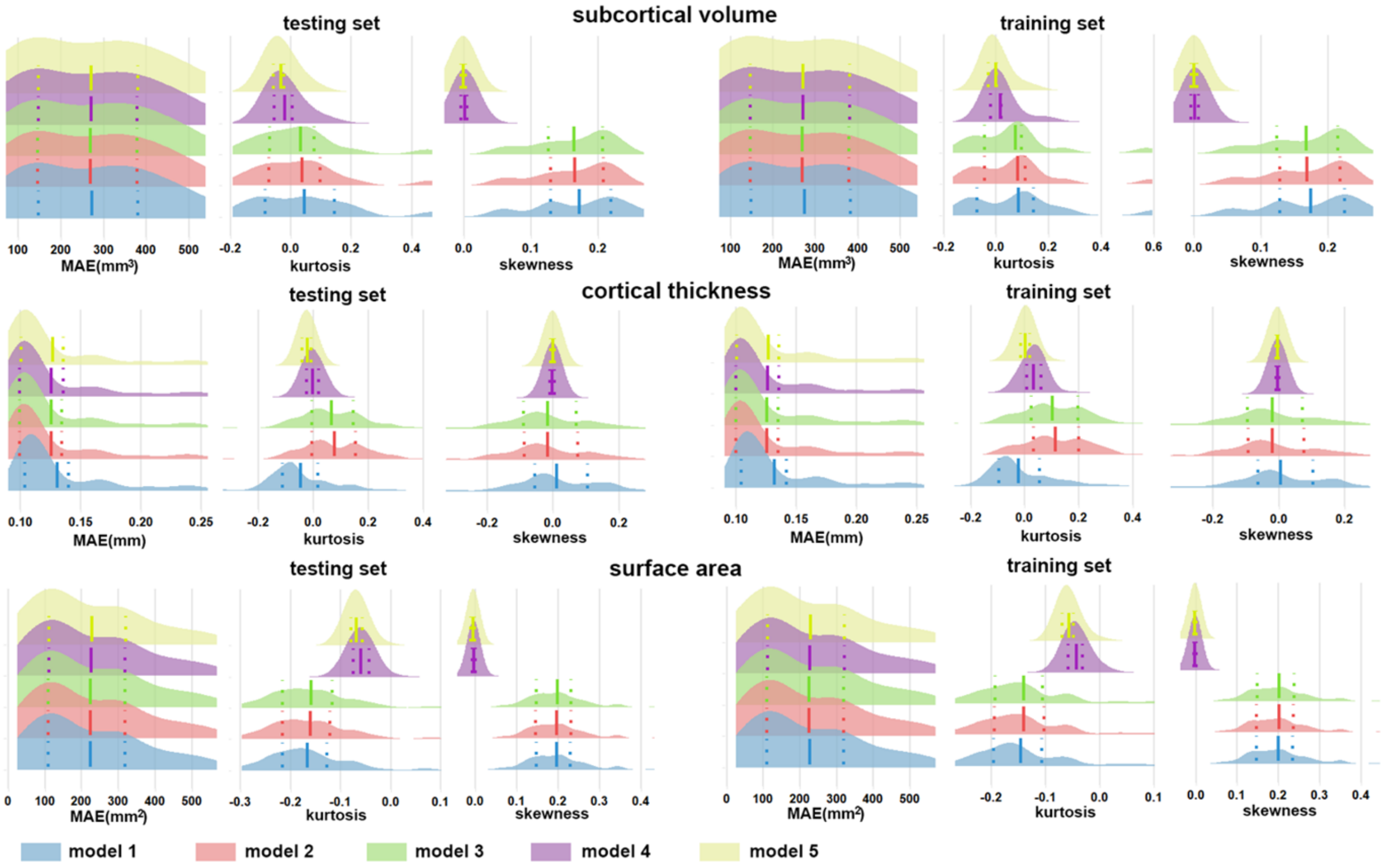

Supplementary Figure S14. **Comparative evaluations of five GAMLSS models in females**. The models were compared in terms of MAE (mean absolute error), kurtosis, and skewness. The model used in our paper is model 4, all other models are from Dinga et al.[12] All models have similar MAE, while model 4 and model 5 performed best in terms of kurtosis and skewness (i.e., for kurtosis and skewness, closer to 0 is better). Since differences in the performance of models 4 and 5 are negligible, the results support our choice of using model 4.

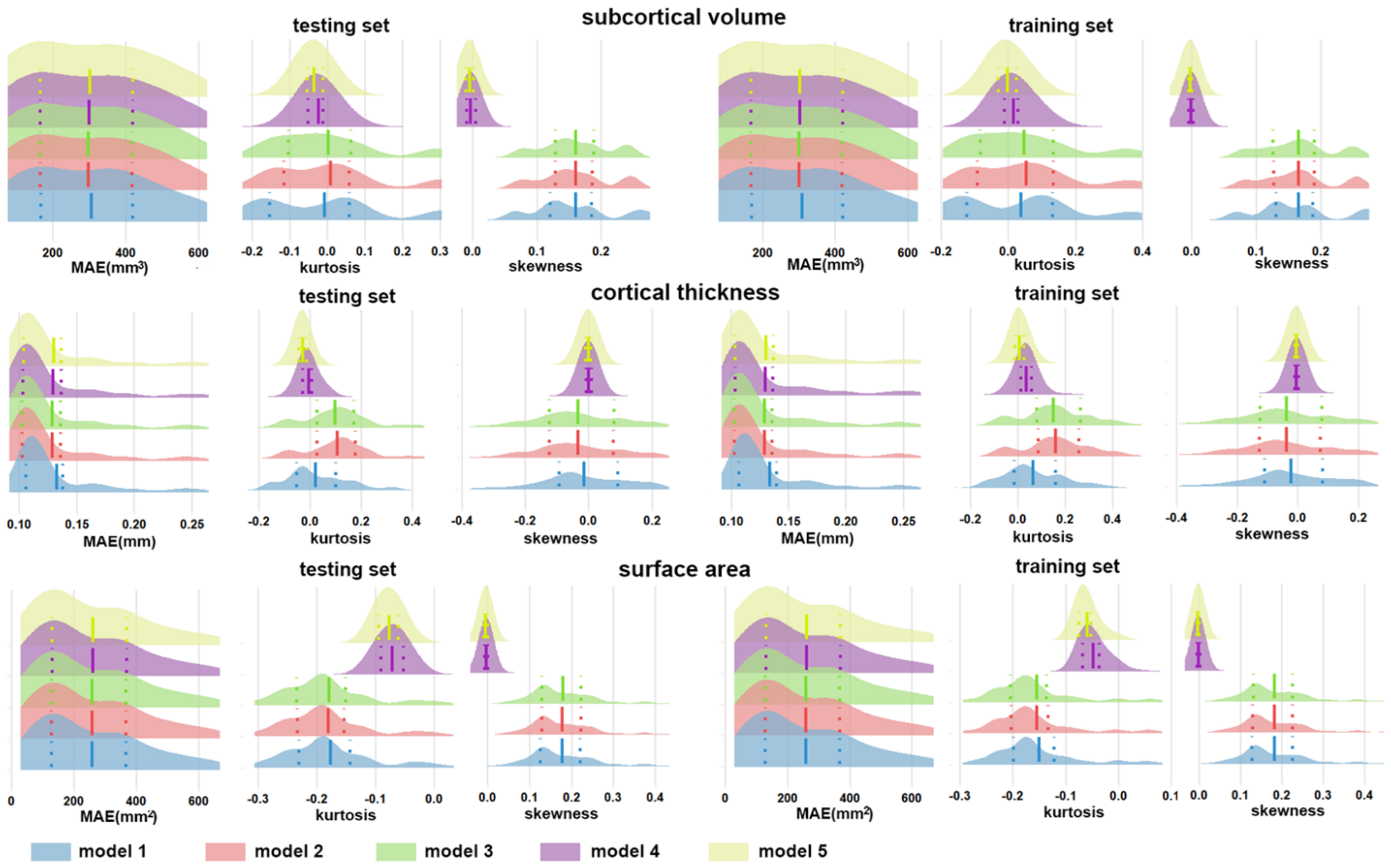

Supplementary Figure S15. **Comparative evaluations of five GAMLSS models in males**. The models were compared in terms of MAE (mean absolute error), kurtosis, and skewness. The model used in our paper is model 4, all other models are from Dinga et al.[12] All models have similar MAE, while model 4 and model 5 performed best in terms of kurtosis and skewness (i.e., for kurtosis and skewness, closer to 0 is better). Since differences in the performance of models 4 and 5 are negligible, the results support our choice of using model 4.

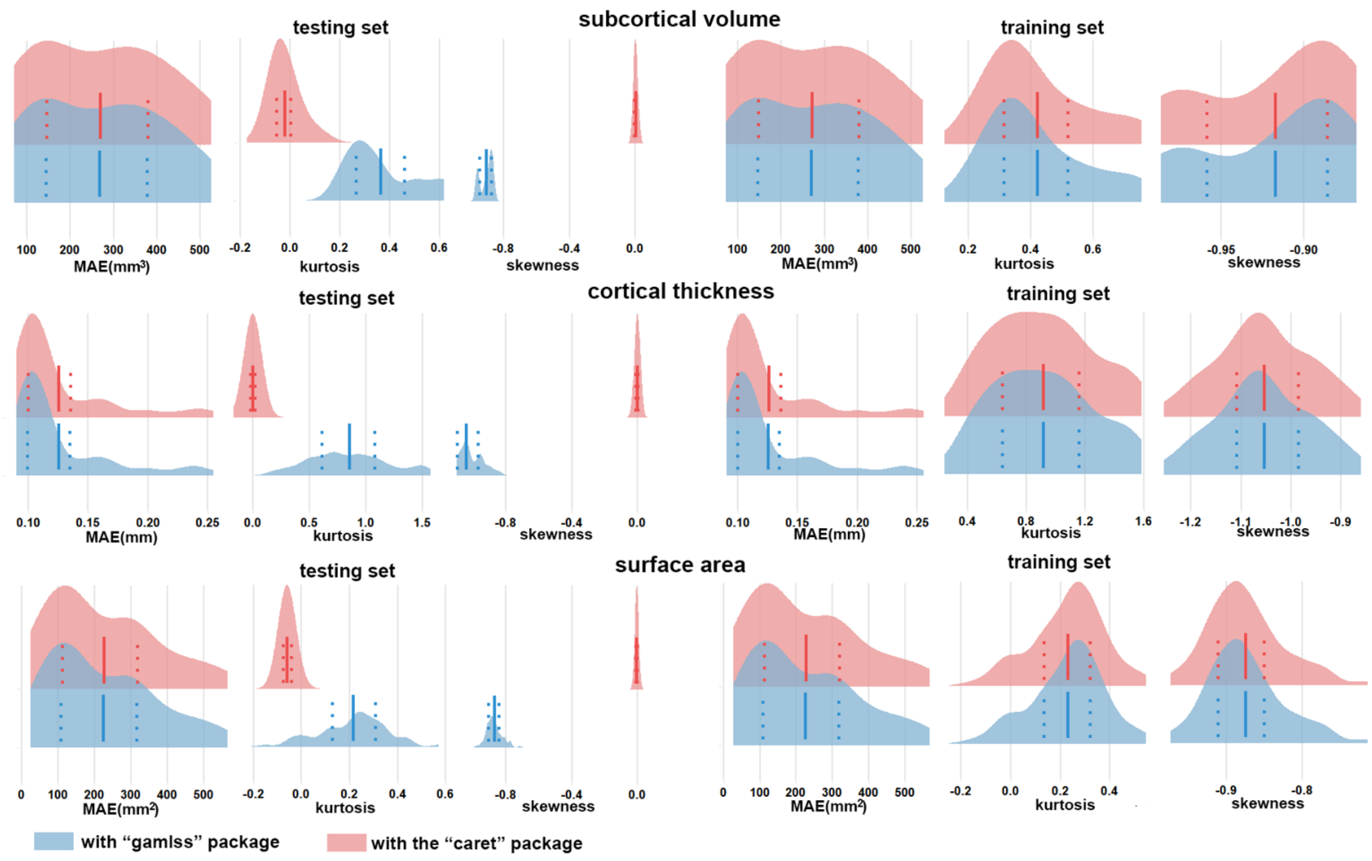

Supplementary Figure S16. Comparative evaluation of GAMLSS models using the "caret" and "gamlss" software in females. GAMLSS models using the "caret" (pink) and "gamlss" (blue) packages in terms of MAE (mean absolute error), kurtosis, and skewness. Model performance in terms of MAE was similar for both packages. The output of the "caret" package used in our paper performed better in terms of the model fit of kurtosis and skewness (for kurtosis and skewness, closer to 0 is better) for the testing set.

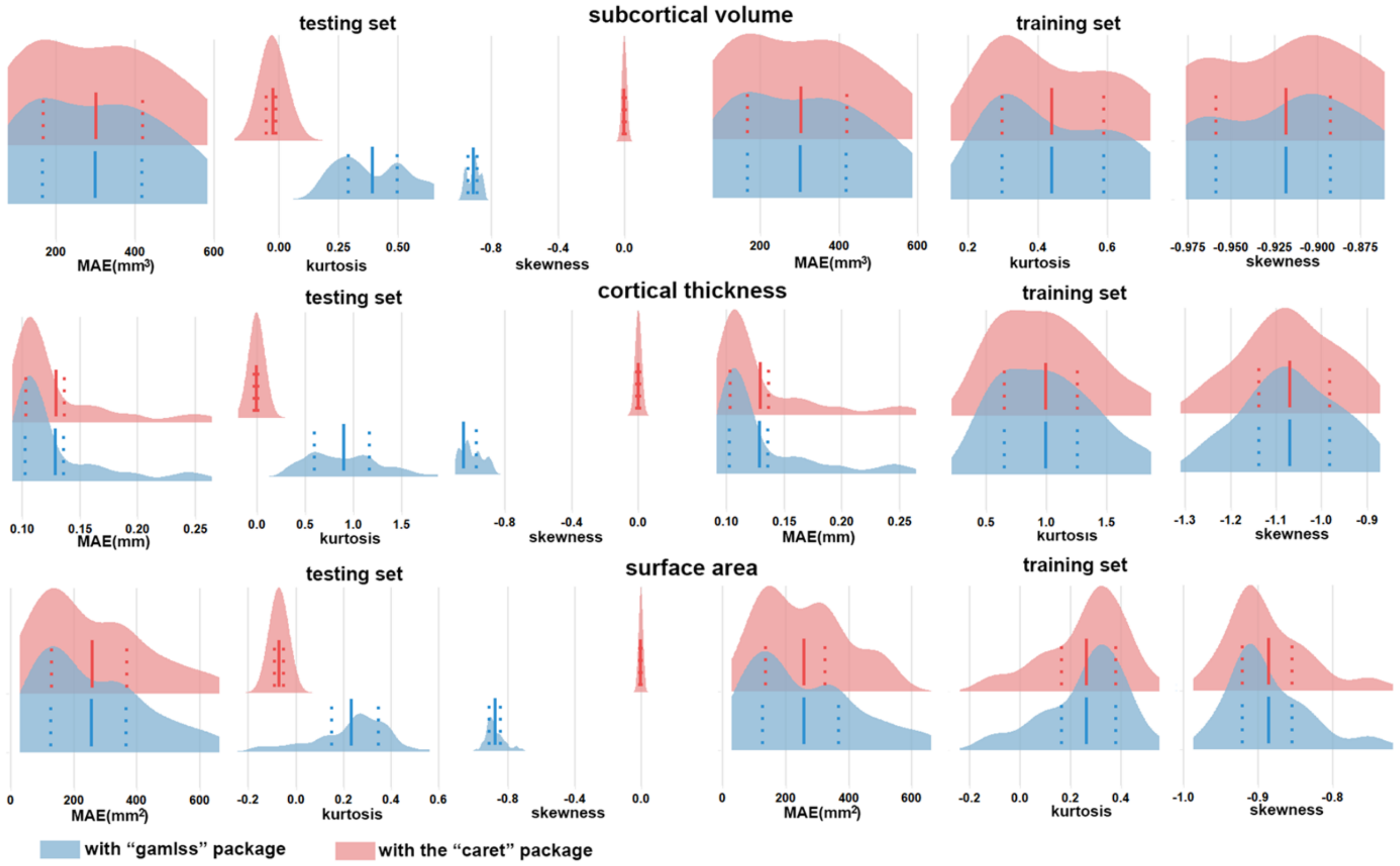

Supplementary Figure S17. Comparative evaluation of GAMLSS models using the "caret" and "gamlss" software in males. GAMLSS models using the "caret" (pink) and "gamlss" (blue) packages in terms of MAE (mean absolute error), kurtosis, and skewness. Model performance in terms of MAE was similar for both packages. The output of the "caret" package used in our paper performed better in terms of the model fit of kurtosis and skewness (for kurtosis and skewness, closer to 0 is better) for the testing set.

S5.4 Sensitivity analyses pertaining to site

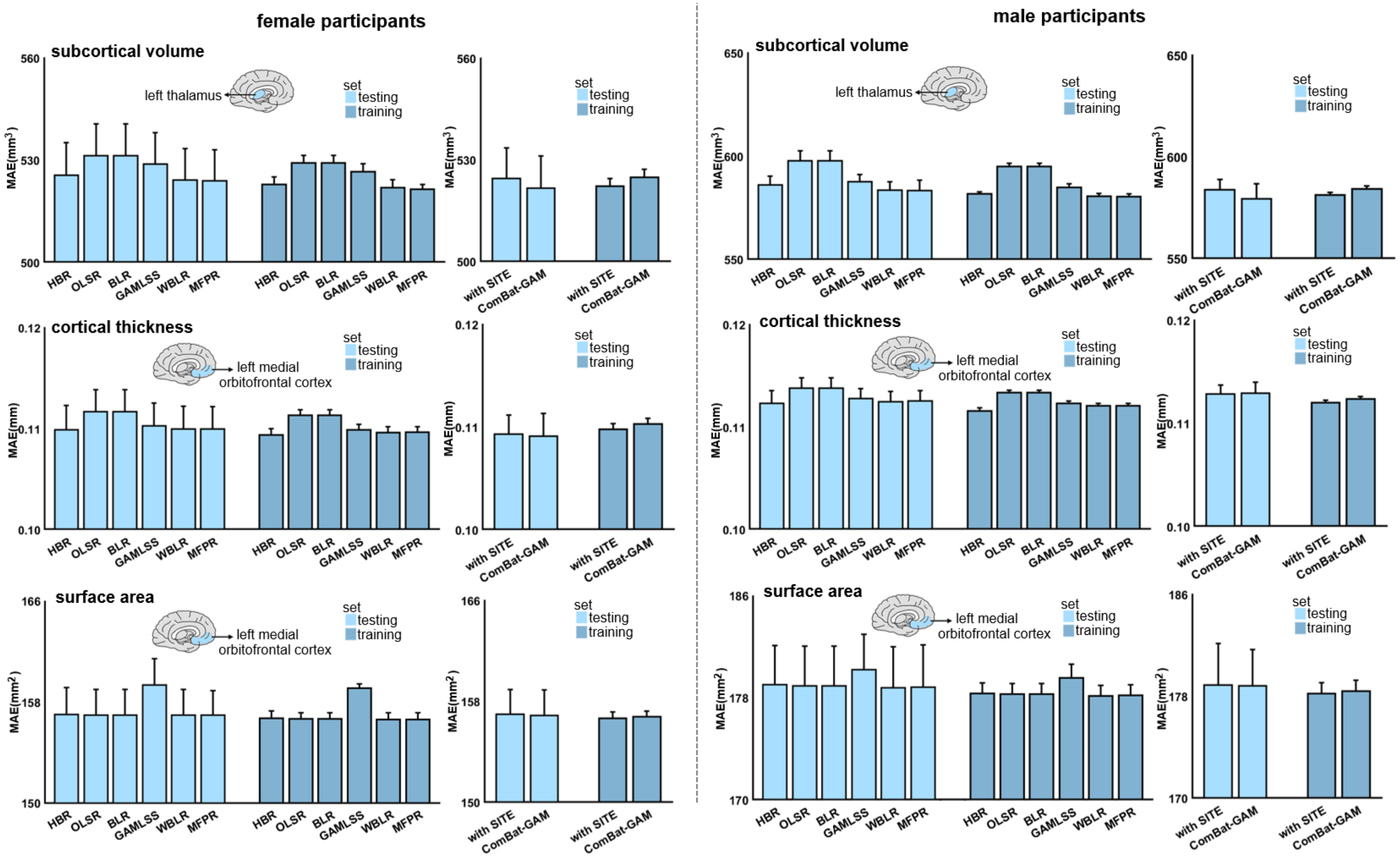

Supplementary Figure S18. Effect of site handling on algorithm performance. The performance of algorithms [Hierarchical Bayesian Regression (HBR); Ordinary Least Squares Regression (OLSR); Bayesian Linear Regression (BLR); Generalized Additive Models for Location, Scale, and Shape (GAMLSS); Warped Bayesian Linear Regression (WBLR); Multivariable Fractional Polynomial Regression (MFPR)] was compared when site was modeled either as a random factor or removed by site harmonisation using ComBat-GAM. The top performing algorithm when the site was used as a random effect was still the MFPR, followed closely by WBLR. Further, the model performance of the MFPR algorithm in terms of MAE was similar regardless of how site was handled.

S6. Clinical relevance of normative brain modeling for mental illness

We tested the relative advantage of the brain regional normative deviation scores (Z-scores) compared to observed neuromorphometric data for the discrimination of healthy individuals from those with early onset psychosis and for the prediction of psychotic symptom severity. To achieve this, we used data from the Human Connectome Project (HCP) Early Psychosis Study which comprises cross-sectional neuroimaging data from 91 individuals with early psychosis and 57 healthy individuals (total sample: 48 females/100 males; age range 16.67-35.67 years). T1-weighted images were downloaded from the HCP repository (https://www.humanconnectome.org/study/human-connectome-project-for-early-psychosis) and preprocessed using FreeSurfer (version 7.1.0) to generate regional brain morphometric data. Then brain regional Z-scores were derived from each of the algorithms examined here. As described in the main manuscript, the classification accuracy of support vector classifiers (SVC) using brain regional Z-scores were compared to that of the classifier using observed data. Predictive accuracy for psychotic symptom severity was assessed using ridge regression models using brain regional Z-scores compared to that of the classifier using observed data.

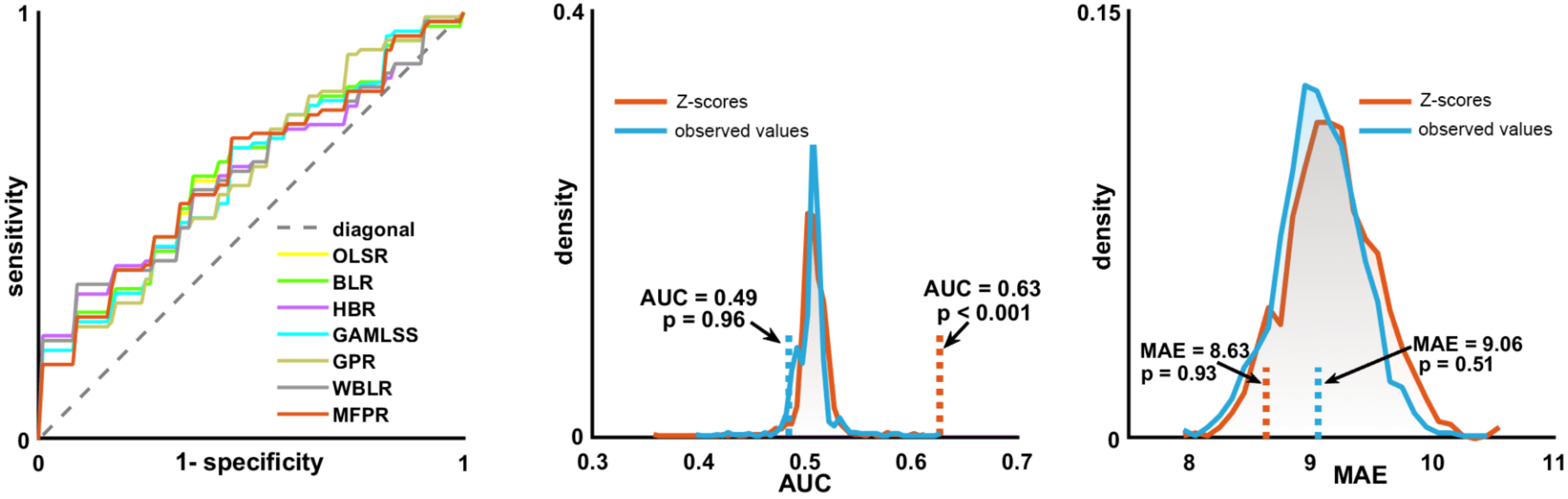

Supplementary Figure S19. Comparative accuracy of diagnostic classification and symptom severity prediction in psychosis using either brain regional Z-scores or observed neuromorphometric data of the Human Connectome Project-Early Psychosis cohort. Left panel: Receiver Operating Characteristic Curves (ROC) of support vector classifiers (SCV) using brain regional Z-scores from each of the algorithms examined here. Middle Panel: Comparative performance in accuracy of diagnostic classification of the SVC using either Z-scores (red) or observed data (blue). Right panel: Predictive accuracy for psychotic symptom severity of ridge regression models using either Z-scores (red) or observed data (blue). In both these panels, two null distributions from the permutation tests are presented: one that illustrates the performance of models using the observed data (distributions with blue outlines) and the other illustrating the performance of models using Z-scores (distributions with red outlines). AUC=area under the curve; MAE=mean absolute error; OLSR=Ordinary Least Squares Regression; BLR=Bayesian Linear Regression; HBR=Hierarchical Bayesian Regression; GAMLSS=Generalized Additive Models for Location, Scale, and Shape; GPR=Gaussian Process Regression; WBLR=Warped Bayesian Linear Regression; MFPR=Multivariable Fractional Polynomial Regression.

S7. List of appendices 2-6 in Excel format

Appendix 2: Supplementary Table S1. Datasets included in the model optimization sample with access information.
Appendix 3: Supplementary Table S2. Performance of the eight algorithms for regional subcortical volume models in females
Appendix 3: Supplementary Table S3. Performance of the eight algorithms for regional subcortical volume models in males
Appendix 3: Supplementary Table S4. Performance of the eight algorithms for regional cortical thickness models in females
Appendix 3: Supplementary Table S5. Performance of the eight algorithms for regional cortical thickness models in males
Appendix 3: Supplementary Table S6. Performance of the eight algorithms for regional cortical surface area models in females
Appendix 3: Supplementary Table S7. Performance of the eight algorithms for regional cortical surface area models in males
Appendix 4: Supplementary Table S8. Performance of MFPR-derived models of regional subcortical volumes in females as a function of explanatory variables
Appendix 4: Supplementary Table S9. Performance of MFPR-derived models of regional subcortical volumes in males as a function of explanatory variables
Appendix 4: Supplementary Table S10. Performance of MFPR-derived models of regional cortical thickness in females as a function of explanatory variables
Appendix 4: Supplementary Table S11. Performance of MFPR-derived models of regional cortical thickness in males as a function of explanatory variables
Appendix 4: Supplementary Table S12. Performance of MFPR-derived models of regional cortical surface area in females as a function of explanatory variables
Appendix 4: Supplementary Table S13. Performance of MFPR-derived models of regional cortical surface area in males as a function of explanatory variables
Appendix 5: Supplementary Table S14. Comparative performance and CPU time of optimized OLSR, BLR, HBR, GPR, GAMLSS, WBLR, and MFPR-derived models for regional subcortical volumes in females
Appendix 5: Supplementary Table S15. Comparative performance and CPU time of optimized OLSR, BLR, HBR, GPR, GAMLSS, WBLR, and MFPR-derived models for regional subcortical volumes in males
Appendix 5: Supplementary Table S16. Comparative performance and CPU time of optimized OLSR, BLR, HBR, GPR, GAMLSS, WBLR, and MFPR-derived models for regional cortical thickness in females
Appendix 5: Supplementary Table S17. Comparative performance and CPU time of optimized OLSR, BLR, HBR, GPR, GAMLSS, WBLR, and MFPR-derived models for regional cortical thickness in males
Appendix 5: Supplementary Table S18. Comparative performance and CPU time of optimized OLSR, BLR, HBR, GPR, GAMLSS, WBLR, and MFPR-derived models for regional cortical surface area in females
Appendix 5: Supplementary Table S19. Comparative performance and CPU time of optimized OLSR, BLR, HBR, GPR, GAMLSS, WBLR, and MFPR-derived models for regional cortical surface area in males
Appendix 6: Supplementary Table S20. Performance of MFPR-derived models of regional subcortical volumes per age bin in females
Appendix 6: Supplementary Table S21. Performance of MFPR-derived models of regional subcortical volumes per age bin in males
Appendix 6: Supplementary Table S22. Performance of MFPR-derived models of regional cortical thickness per age bin in females
Appendix 6: Supplementary Table S23. Performance of MFPR-derived models of regional cortical thickness per age bin in males
Appendix 6: Supplementary Table S24. Performance of MFPR-derived models of regional cortical surface area per age bin in females
Appendix 6: Supplementary Table S25. Performance of MFPR-derived models of regional cortical surface area per age bin in males

Supplementary references

1. Ching CRK, Gutman BA, Sun D, et al. Mapping subcortical brain alterations in 22q11.2 deletion syndrome: effects of deletion size and convergence with idiopathic neuropsychiatric illness. *Am J Psychiatry* 2020; **177**: 589–600.
2. Jalbrzikowski M, Hayes RA, Wood SJ, Nordholm D, Zhou JH, Fusar-Poli P, et al. Association of structural magnetic resonance imaging measures with psychosis onset in individuals at clinical high risk for developing psychosis: an ENIGMA working group mega-analysis. *JAMA Psychiatry* 2021; **78**: 753–66.
3. Schmaal L, Veltman DJ, van Erp TG, et al. Subcortical brain alterations in major depressive disorder: findings from the ENIGMA Major Depressive Disorder working group. *Mol Psychiatry* 2016; **21**: 806–12.
4. Schmaal L, Hibar DP, Sämann PG, et al. Cortical abnormalities in adults and adolescents with major depression based on brain scans from 20 cohorts worldwide in the ENIGMA Major Depressive Disorder working group. *Mol Psychiatry* 2017; **22**: 900–9.
5. Sun D, Ching CRK, Lin A, et al. Large-scale mapping of cortical alterations in 22q11.2 deletion syndrome: convergence with idiopathic psychosis and effects of deletion size. *Mol Psychiatry* 2020; **25**: 1822–34.
6. van Erp TG, Hibar DP, Rasmussen JM, et al. Subcortical brain volume abnormalities in 2028 individuals with schizophrenia and 2540 healthy controls via the ENIGMA consortium. *Mol Psychiatry* 2016; **21**: 547–53.
7. van Erp TGM, Walton E, Hibar DP, et al. Cortical brain abnormalities in 4474 individuals with schizophrenia and 5098 control subjects via the enhancing neuro imaging genetics through meta-analysis (ENIGMA) Consortium. *Biol Psychiatry* 2018; **84**: 644–54.
8. Hagler DJ, Hatton S, Cornejo MD, et al. Image processing and analysis methods for the Adolescent Brain Cognitive Development Study. *Neuroimage* 2019; **202**: 116091.
9. Klapwijk ET, van de Kamp F, van der Meulen M, Peters S, Wierenga LM. Qoala-T: a supervised-learning tool for quality control of FreeSurfer segmented MRI data. *Neuroimage* 2019; **189**: 116–29.
10. Monereo-Sánchez J, de Jong JJA, Drenthen GS, et al. Quality control strategies for brain MRI segmentation and parcellation: practical approaches and recommendations - insights from the Maastricht study. *NeuroImage* 2021; **237**: 118174.
11. Rosen AFG, Roalf DR, Ruparel K, et al. Quantitative assessment of structural image quality. *Neuroimag*e 2018; **169**: 407–418.
12. Dinga R, Fraza CJ, Bayer JMM, Kia SM, Beckmann CF, Marquand AF. Normative modeling of neuroimaging data using generalized additive models of location scale and shape. *bioRxiv* 2021; published online June 14. doi: https://doi.org/10.1101/2021.06.14.448106 (preprint).

ENIGMA Lifespan working group authors and affiliations

Prof Ingrid Agartz MD[1], Prof Philip Asherson MD[2], Rosa Ayesa-Arriola PhD[3], Nerisa Banaj PhD[4], Prof Tobias Banaschewski PhD[5], Prof Sarah Baumeister PhD[5], Prof Alessandro Bertolino MD[6,7], Prof Stefan Borgwardt MD[8], Josiane Bourque M.Sc[9], Prof Daniel Brandeis PhD[5], Prof Alan Breier MD[10], Prof Jan K Buitelaar MD[11], Dara M Cannon PhD[12], Simon Cervenka MD[13], Prof Patricia J Conrod PhD[14], Prof Benedicto Crespo-Facorro MD[15,16], Prof Christopher G Davey MD[17], Prof Lieuwe de Haan MD[18], Greig I de Zubicaray PhD[19], Annabella Di Giorgio MD[20], Prof Thomas Frodl MD[21,22], Patricia Gruner PhD[23], Prof Raquel E Gur MD[24,25], Prof Ruben C Gur PhD[24,25], Prof Ben J Harrison PhD[26], Prof Sean N Hatton PhD[27], Prof Ian Hickie MD[28], Fleur M Howells PhD[29], Chaim Huyser PhD[30], Prof Terry L Jernigan PhD[31], Jiyang Jiang PhD[32], Prof John A Joska MD[33], Prof René S Kahn MD[34], Prof Andrew J Kalnin MD[35], Nicole A Kochan PhD[32], Sanne Koops PhD[36], Prof Jonna Kuntsi PhD[2], Prof Jim Lagopoulos PhD[37], Luisa Lazaro MD[38,39], Irina S Lebedeva DrSci[40], Christine Lochner PhD[41], Prof Nicholas G Martin PhD[42], Prof Bernard Mazoyer PhD[43], Prof Brenna C McDonald PsyD[44], Prof Colm McDonald PhD[45], Prof Katie L McMahon PhD[46], Prof Sarah Medland PhD[47], Amirhossein Modabbernia MD[34], Benson Mwangi PhD[48], Prof Tomohiro Nakao PhD[49], Prof Lars Nyberg PhD[50], Fabrizio Piras PhD[4], Maria J Portella PhD[51,39,52], Prof Jiang Qiu PhD[53], Joshua L Roffman MD[54], Prof Perminder S Sachdev MD[32], Nicole Sanford PhD[55], Theodore D Satterthwaite MD[56], Prof Andrew J Saykin PsyD[57], Carl M Sellgren MD[58], Prof Kang Sim MD[59], Prof Jordan W Smoller MD[60], Prof Jair C Soares MD[61], Prof Iris E Sommer MD[36], Gianfranco Spalletta PhD[4], Prof Dan J Stein MD[62], Sophia I Thomopoulos BA[63], Alexander S Tomyshev MSc[40], Diana Tordesillas-Gutiérrez PhD[64], Prof Julian N Trollor MD[65], Prof Dennis van 't Ent PhD[66], Prof Odile A van den Heuvel MD[67], Theo GM van Erp PhD[68], Prof Neeltje EM van Haren PhD[69], Daniela Vecchio PhD[4], Prof Dick J Veltman MD[70], Prof Yang Wang PhD[71], Prof Bernd Weber MD[72], Dongtao Wei PhD[53], Prof Wei Wen PhD[32], Prof Lars T Westlye PhD[73], Prof Steven CR Williams PhD[74], Prof Margaret J Wright PhD[75], Mon-Ju Wu PhD[76], Kevin Yu PhD[55]

[1]University of Oslo, PB 85 Vinderen, 0319, Oslo, Norway

[2]Social, Genetic and Developmental Psychiatry Centre, Institute of Psychiatry, Psychology and Neuroscience, King's College London, De Crespigny Park, London SE5 8AF, UK

[3]Department of Psychiatry, Marqués de Valdecilla University Hospital, Valdecilla Biomedical Research Institute (IDIVAL), s/n, Calle Cardenal Herrera Oria, 39011, Santander, Cantabria, Spain

[4]Neuropsychiatry Laboratory, Department of Clinical and Behavioral Neurology, IRCCS Santa Lucia Foundation, Via Ardeatina 306, 00179, Rome, Italy

[5]Department of Child and Adolescent Psychiatry and Psychotherapy, Central Institute of Mental Health, Medical Faculty Mannheim, University of Heidelberg, J5, 68159, Mannheim, Germany

[6]Department of Translational Biomedicine and Neuroscience, University of Bari Aldo Moro, Piazza Giulio Cesare, 11, 70124, Bari, Italy

[7]Amsterdam Reproduction & Development research institute, Amsterdam, The Netherlands

[8]Department of Psychiatry and Psychotherapy, University of Lübeck, Ratzeburger Allee 160, 23562, Lübeck, Germany

[9]Department of Psychiatry, University of Pennsylvania Perelman School of Medicine, 3400 Spruce Street, 10th Floor, Gates Pavilion, Philadelphia, PA, 19104, USA

[10]Department of Psychiatry, Indiana University School of Medicine, 320 W. 15th St, Indianapolis, IN, 46202, USA

[11]Department of Cognitive Neuroscience, Donders Institute for Brain, Cognition and Behaviour, Radboud university medical center (Radboudumc), P.O. box 9101 (internal post 204), 6500 HB, Nijmegen, The Netherlands

[12]Clinical Neuroimaging Laboratory, Galway Neuroscience Centre, College Medicine, Nursing & Health Sciences, University of Galway, 1021 Human Biology Building, University Road, H91 TK33, Galway, Ireland

[13]Department of Medical Sciences, Psychiatry, Uppsala University, Uppsala University Hospital, Ing10, 751 85, Uppsala, Sweden

[14]Department of Psychiatry and Addiction, Université de Montréal, CHU Ste Justine, Pavillon Roger-Gaudry, 2900, boulevard Édouard-Montpetit, Montréal (Québec), H3T 1J4, Canada

[15]Department of Psychiatry, School of Medicine, Virgen del Rocio University, University of Seville, Instituto de Biomedicina de Sevilla (IbiS), C. Antonio Maura Montaner, 41013, Sevilla, Spain

[16]Centro de Investigación Biomédica en Red de Salud Mental (CIBERSAM), Spain
[17]Department of Psychiatry, The University of Melbourne, Level 3, Alan Gilbert Building, 161 Barry Street, Carlton, VIC, 3053, Australia
[18]Department of Psychiatry, Amsterdam UMC, Meibergdreef 5, 1105 AZ, Amsterdam, The Netherlands
[19]School of Psychology and Counselling, Queensland University of Technology, Brisbane, QLD, 4059, Australia
[20]Department of Mental Health and Addictions, ASST Papa Giovanni XXIII, Piazza OMS 1, 24127, Bergamo, Italy
[21]Department of Psychiatry and Psychotherapy, Otto von Guericke University Magdeburg (OVGU), Universitätspl. 2, 39106, Magdeburg, Germany
[22]Department of Psychiatry, Psychotherapy and Psychosomatics, University Hospital, RWTH Aachen, Pauwelsstraße 30, 52074, Aachen, Germany
[23]Department of Psychiatry, Yale University, 300 George St #901, New Haven, CT, 6511, USA
[24]Department of Psychiatry, Neurodevelopment and Psychosis Section, University of Pennsylvania Perelman School of Medicine, 3400 Spruce Street, 10th Floor, Gates Pavilion, Philadelphia, PA, 19104, USA
[25]Penn-CHOP Lifespan Brain Institute, Children's Hospital of Philadelphia and Penn Medicine, University of Pennsylvania, 3401 Civic Center Blvd, Philadelphia, PA, 19104, USA
[26]Melbourne Neuropsychiatry Centre, Department of Psychiatry, The University of Melbourne, Level 3, Alan Gilbert Building, 161 Barry Street, Carlton, VIC, 3053, Australia
[27]Center for Multimodal Imaging and Genetics, University of California San Diego, Floor 4 West, 9444 Medical Ctr Dr, La Jolla, CA, 92037, USA
[28]Brain and Mind Centre, The University of Sydney, Level 4, 94 Mallett Street, Camperdown, NSW, 2050, Australia
[29]Department of Psychiatry and Mental Health, University of Cape Town, Anzio Road, Observatory, Cape Town, 7925, South Africa
[30]Levvel, Academic Center for Child and Adolescent Psychiatry, Meibergdreef 5, 1105 AZ, Amsterdam, The Netherlands
[31]Center for Human Development, University of California San Diego, 9500 Gilman Dr., La Jolla, CA, 92093-0115, USA
[32]Centre for Healthy Brain Ageing (CHeBA), Discipline of Psychiatry & Mental Health, University of New South Wales Sydney, Level 1, AGSM Building, Gate 11, UNSW, Sydney, NSW, 2052, Australia
[33]HIV Mental Health Research Unit, Department of Psychiatry and Mental Health, Neuroscience Institute, Groote Schuur Hospital, University of Cape Town, Main Rd, Observatory, Cape Town, 7935, South Africa
[34]Department of Psychiatry, Icahn School of Medicine at Mount Sinai, One Gustave L. Levy Place, New York, NY, 10029, USA
[35]Department of Radiology, The Ohio State University College of Medicine, 395 W. 12th Ave, 4th Floor, Columbus, OH, 43210, USA
[36]Department of Psychiatry, University Medical Center Groningen (UMCG), Rijksuniversiteit Groningen, Antonius Deusinglaan 2, 9713 AW, Groningen, The Netherlands
[37]Thompson Institute, University of the Sunshine Coast, 12 Innovation Pwy, Birtinya, QLD, 4575, Australia
[38]Department of Child and Adolescent Psychiatry and Psychology, Hospital Clínic of Barcelona, IDIBAPS, University of Barcelona, C/ Villarroel, 170, 08036, Barcelona, Spain
[39]Centro de Investigación Biomédica en Red de Salud Mental (CIBERSAM), Madrid, Spain
[40]Mental Health Research Center, 34 Kashirskoe Schosse, 115522, Moscow, Russia
[41]SAMRC Unit on Risk and Resilience in Mental Disorders, Department of Psychiatry, Stellenbosch University, Francie Van Zijl Dr, Parow, Cape Town, 7505, South Africa
[42]QIMR Berghofer Medical Research Institute, 300 Herston Rd, Herston, QLD, 4006, Australia
[43]Groupe d'Imagerie Neurofonctionnelle - Institut des Maladies Neurodégénératives, Université de Bordeaux, CNRS UMR 5293, Bordeaux, France
[44]Department of Radiology and Imaging Sciences, Indiana University School of Medicine, 355 W. 16th St., GH Suite 4100, Indianapolis, IN, 46220, USA
[45]Centre for Neuroimaging & Cognitive Genomics (NICOG), Galway Neuroscience Centre, University of Galway, University Road, H91 TK33, Galway, Ireland
[46]Queensland University of Technology, 2 George St, Brisbane City, QLD, 4000, Australia
[47]Psychiatric Genetics, QIMR Berghofer Medical Research Institute, 300 Herston Rd, Herston, Queensland, Australia, Herston, QLD, 4006, Australia

[48]Center of Excellence on Mood Disorders, Louis A. Faillace, MD, Department of Psychiatry and Behavioral Sciences, McGovern Medical School, The University of Texas Health Science Center at Houston, 1942 East Rd, Office 3130, Houston, TX, 77055, USA
[49]Department of Neuropsychiatry, Kyushu University, 3-1-1, Maidashi, Higashi-ku, Fukuoka City, 812-8582, Japan
[50]Radiation Sciences & IMB, Umeå University, Linnaeus v 15, 901 87, Umeå, Sweden
[51]Mental Health Research Group, Institut d'Investigació Biomèdica Sant Pau (IIB SANT PAU), Hospital de la Santa Creu i Sant Pau, c/ Sant Quintí 77, 08041, Barcelona, Spain
[52]Department of Psychiatry and Forensic Medicine, Universitat Autònoma de Barcelona (UAB), Plaça Cívica, 08193, Barcelona, Spain
[53]Faculty of Psychology, Southwest University, Tiansheng Road 2#, Chongqing, 400715, China
[54]Massachusetts General Hospital and Harvard Medical School, 149 13th St, Room 2606, Charlestown, MA, 02129, USA
[55]Djavad Mowafagian Centre for Brain Health, University of British Columbia, 2215 Wesbrook Mall, Vancouver, BC, V6T 1Z3, Canada
[56]Penn Lifespan Informatics and Neuroimaging Center, University of Pennsylvania Perelman School of Medicine, Richards Medical Labs, 3600 Spruce Street, Philadelphia, PA, 19104, USA
[57]Indiana Alzheimer's Disease Research Center and Center for Neuroimaging, Indiana University School of Medicine, 355 West 16th Street, Indianapolis, IN, 46202, USA
[58]Department of Physiology and Pharmacology, Karolinska Institutet, Solnavägen 9, 171 65, Solna, Sweden
[59]Institute of Mental Health, 10 Buangkok View, 539747, Singapore
[60]Center for Precision Psychiatry, Department of Psychiatry, Massachusetts General Hospital, 185 Cambridge St. 6th FL, Boston, MA, 02114, USA
[61]Center of Excellence on Mood Disorders, Louis A. Faillace, MD, Department of Psychiatry and Behavioral Sciences, McGovern Medical School, The University of Texas Health Science Center at Houston, 1941 East Rd, Office 3210, Houston, TX, 77054, USA
[62]SAMRC Unit on Risk & Resilience in Mental Disorders, Department of Psychiatry & Neuroscience Institute, University of Cape Town, Groote Schuur Hospital, Anzio Rd, Observatory, Cape Town, 7925, South Africa
[63]Mark and Mary Stevens Neuroimaging and Informatics Institute, Keck School of Medicine of USC, University of Southern California, 4676 Admiralty Way, Suite 200, Marina del Rey, CA, 90292, USA
[64]Instituto de Física de Cantabria (CSIC-UC), Edificio Juan Jordá. Av. de los Castros, Santander E-39005, Cantabria, Spain
[65]Department of Developmental Disability Neuropsychiatry, Discipline of Psychiatry and Mental Health, University of New South Wales Sydney, Room 241, Level 2, Biolink Building E25, Sydney, NSW, 2052, Australia
[66]Netherlands Twin Register, Department of Biological Psychology, Vrije Universiteit, Amsterdam, Van der Boechorststraat 7-9, 1081BT, Amsterdam, The Netherlands
[67]Department of Psychiatry, Amsterdam UMC, P.O. box 7057, 1007 MB, Amsterdam, The Netherlands
[68]Department of Psychiatry and Human Behavior, University of California Irvine, Irvine, CA, 92697, USA
[69]Department of Child and Adolescent Psychiatry/Psychology, Erasmus Medical Centre - Sophia Children's Hospital, Wytemaweg 8, KP-2, 3015CN, Rotterdam, The Netherlands
[70]Department of Psychiatry, Amsterdam UMC, location VUMC, P.O. box 7057, 1007 MB, Amsterdam, The Netherlands
[71]Department of Radiology, Medical College of Wisconsin, 8701 Watertown Plank Road, Milwaukee, WI, 53226, USA
[72]Institute of Experimental Epileptology and Cognition Research, University Hospital Bonn, Venusberg-Campus 1, 53127, Bonn, Germany
[73]Department of Psychology, University of Oslo, P.O. box 1094 Blindern, 0317, Oslo, Norway
[74]Department of Neuroimaging, Institute of Psychiatry, Psychology & Neuroscience, King's College London, Denmark Hill, SE5 8AF, London, UK
[75]Queensland Brain Institute, University of Queensland, QBI Building 79, University of Queensland, St Lucia, Queensland, 4067, Australia
[76]Center of Excellence on Mood Disorders, Louis A. Faillace, MD, Department of Psychiatry and Behavioral Sciences, McGovern Medical School, The University of Texas Health Science Center at Houston, 1941 East Rd, Office 3130, Houston, TX, 77054, USA
